# Harnessing accurate non-homologous end joining for efficient precise deletion in CRISPR/Cas9-mediated genome editing

**DOI:** 10.1186/s13059-018-1518-x

**Published:** 2018-10-19

**Authors:** Tao Guo, Yi-Li Feng, Jing-Jing Xiao, Qian Liu, Xiu-Na Sun, Ji-Feng Xiang, Na Kong, Si-Cheng Liu, Guo-Qiao Chen, Yue Wang, Meng-Meng Dong, Zhen Cai, Hui Lin, Xiu-Jun Cai, An-Yong Xie

**Affiliations:** 1 Department of General Surgery, Innovation Center for Minimally Invasive Techniques and Devices, Sir Run Run Shaw Hospital, Zhejiang University School of Medicine, Zhejiang, 310019 Hangzhou China; 2 Institute of Translational Medicine, Zhejiang University School of Medicine, Zhejiang, 310029 Hangzhou China; 3 Department of General Surgery, Chongqing General Hospital, Chongqing, 400013 China; 4 Multiple Myeloma Treatment Center & Bone Marrow Transplantation Center, The First Affiliated Hospital, Zhejiang University School of Medicine, Zhejiang, 310003 Hangzhou China

**Keywords:** Paired gRNAs, CRISPR/Cas9, Accurate NHEJ, Templated insertions, Precise deletion, Knockout, Targeted in-frame deletion, Genome editing

## Abstract

**Background:**

Many applications of CRISPR/Cas9-mediated genome editing require Cas9-induced non-homologous end joining (NHEJ), which was thought to be error prone. However, with directly ligatable ends, Cas9-induced DNA double strand breaks may be repaired preferentially by accurate NHEJ.

**Results:**

In the repair of two adjacent double strand breaks induced by paired Cas9-gRNAs at 71 genome sites, accurate NHEJ accounts for about 50% of NHEJ events. This paired Cas9-gRNA approach underestimates the level of accurate NHEJ due to frequent + 1 templated insertions, which can be avoided by the predefined Watson/Crick orientation of protospacer adjacent motifs (PAMs). The paired Cas9-gRNA strategy also provides a flexible, reporter-less approach for analyzing both accurate and mutagenic NHEJ in cells and in vivo, and it has been validated in cells deficient for *XRCC4* and in mouse liver. Due to high frequencies of precise deletions of defined “3n”-, “3n + 1”-, or “3n + 2”-bp length, accurate NHEJ is used to improve the efficiency and homogeneity of gene knockouts and targeted in-frame deletions. Compared to “3n + 1”-bp, “3n + 2”-bp can overcome + 1 templated insertions to increase the frequency of out-of-frame mutations. By applying paired Cas9-gRNAs to edit MDC1 and key 53BP1 domains, we are able to generate predicted, precise deletions for functional analysis. Lastly, a Plk3 inhibitor promotes NHEJ with bias towards accurate NHEJ, providing a chemical approach to improve genome editing requiring precise deletions.

**Conclusions:**

NHEJ is inherently accurate in repair of Cas9-induced DNA double strand breaks and can be harnessed to improve CRISPR/Cas9 genome editing requiring precise deletion of a defined length.

**Electronic supplementary material:**

The online version of this article (10.1186/s13059-018-1518-x) contains supplementary material, which is available to authorized users.

## Background

The CRISPR/Cas9 technology has revolutionized many fields of research and commercial development in basic science, medicine, and agriculture [1, 2]. As one of the most common applications, genome editing mediated by CRISPR/Cas9 usually requires targeted induction of a DNA double strand break (DSB) and its repair at a target genome site [3–5]. Manipulation of such DSB induction and repair yields opportunities for optimizing genome editing for a variety of efficient and precise genome modifications [1, 6, 7].

In mammalian cells, Cas9-induced DSBs are repaired largely by non-homologous end joining (NHEJ) and partly by homology-directed repair (HDR) that includes gene conversion, microhomology-mediated end joining (MMEJ), and single strand annealing (SSA) [1, 6]. As an error prone repair pathway, NHEJ generates a high frequency of insertions or deletions (indels) at the repair junctions. Despite potential bias induced by end resection and use of microhomologies during end joining, generation of indels is rather random [7–10]. Thus, these indels are heterogeneous in size and in DNA context. While Cas9-induced indels could inactivate a gene by frame-shift or disruption of key elements, generating gene knockouts, many remain in-frame. As a result, the knockout efficiency is low and additional effort has to be made in identification of knockout clones.

Previously, we and others have shown that a majority (up to 75%) of I-SceI-induced DSBs are rejoined without error in mammalian cells proficient for the classic NHEJ pathway [9–15]. It is expected that Cas9-induced blunt ends can also be readily and accurately re-ligated [16]. In fact, repair profiling of two Cas9-induced DSBs that are hundreds or thousands of base pairs (bp) apart at a few loci has revealed that a portion of distal ends of these two DSBs are accurately ligated [4, 17–23]. This suggests that many Cas9-induced DSBs, if not most, are repaired by accurate NHEJ. Knowledge of accurate NHEJ in repair of Cas9-induced DSBs could help yield a strategy to shift NHEJ away from accurate NHEJ or even to harness accurate NHEJ to generate precise indels of defined length, promoting CRISPR/Cas9-mediated gene knockouts. It is therefore important to systematically examine accurate NHEJ and determine its level in repair of Cas9-induced DSBs.

Genome editing often requires precise deletion of a particular DNA region with defined length. Continuing expansion of CRISPR/Cas9 protospacer adjacent motif (PAM) compatibility will further increase the application spectrum for precise deletion in genome editing [24–26]. Currently, precise deletion can be achieved by MMEJ-mediated insertion or HDR-mediated replacement with a donor DNA containing predefined deletion [1, 6, 7]. However, these two approaches are usually inefficient, and MMEJ-mediated insertion is error prone, limiting their application for precise deletion. A dual nuclease was recently constructed to generate precise deletions of 33 to 36 bp at frequencies of up to 40% [27]. In order to expand the range and efficiency of precise deletion and the scope of its application in genome editing, new approaches are still needed.

Here, we used paired gRNAs to guide *Streptococcus pyogenes* Cas9 to induce two concurrent DSBs that are 23–148 bp apart at 70 endogenous genome sites in mouse and human cells and 1439 bp apart at one site. Repair profiling revealed that NHEJ is inherently accurate in the repair of Cas9-induced DSBs. By identifying and subsequently controlling the factors that influence accurate NHEJ in repair of two close and concurrent DSBs induced by paired Cas9-gRNAs, we were able to increase precise out-of-frame or in-frame deletions of defined length and improve CRISPR/Cas9-mediated genome editing, including gene knockouts and targeted in-frame deletions. In addition, this paired Cas9-gRNA approach was validated as a flexible and reliable reporterless assay for both accurate and mutagenic NHEJ in cells and in vivo.

## Results

### Repair of CRISPR/Cas9-induced DSBs by NHEJ is inherently accurate

**Fig. 1:**
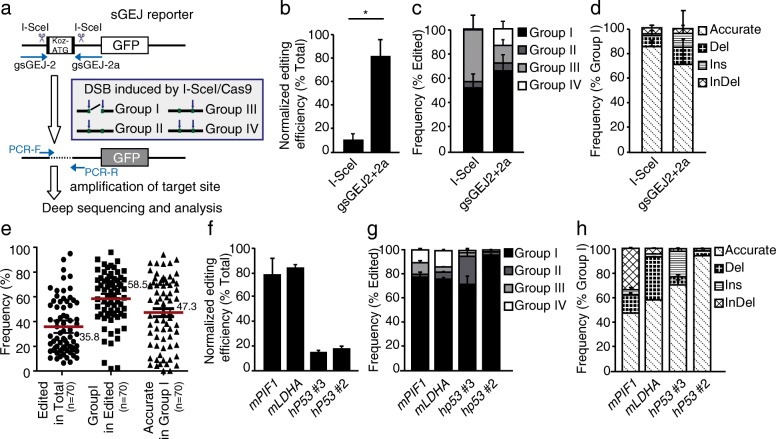
Repair of Cas9-induced DSBs by NHEJ is inherently accurate. **a** The sGEJ reporter. NHEJ repair of paired DSBs induced by I-SceI or Cas9-gRNAs are indicated. Repair products are divided into four groups based on DSBs induced at either or both target sites by I-SceI or Cas9-gRNA in the reporter. Groups I, II, III, and IV represent, respectively, NHEJ for DNA cleavage simultaneously at both target sites, only at the first target site, only at the second target site, and individually at two target sites as indicated. **b–d** Analysis of I-SceI- or paired gRNA-guided Cas9-induced NHEJ in the sGEJ reporter. The normalized editing efficiency (**b**) was calculated as ratios of edited events to total reads and normalized by transfection efficiency. The frequency of group I, group II, group III, and group IV (**c**) was calculated as ratios of reads from each group to total edited reads. The frequency of accurate NHEJ, deletion, insertion, and InDel (**d**) was calculated as ratios of reads from each category in group I to total group I reads. Bars represent the mean ± standard deviation (SD) of three independent experiments. For normalized editing efficiency (**b**), Student’s paired *t*-test between I-SceI and gsGEJ2 + 2a *P = 0.025. **e** Analysis of NHEJ induced by paired Cas9-gRNAs at 70 endogenous genome sites from mouse and human cells. The editing efficiency without transfection efficiency, the frequency of group I in edited events, and the frequency of accurate NHEJ in group I events from each site were analyzed. Bars represent the mean ± SD. **f–h** Analysis of NHEJ induced by paired Cas9-gRNA at *mPIF1*, *mLDHA*, and two *hp53* sites. The normalized editing efficiency (**f**), the frequency of each group in edited events (**g**), and the frequency of each category in group I events (**h**) were calculated. Bars represent the mean ± SD of three independent experiments

Previously, we developed a reporter to analyze NHEJ in mouse embryonic stem (ES) cells and found that I-SceI-induced NHEJ is mostly accurate [11, 28]. In this reporter, no wild-type GFP can be synthesized in cells due to the upstream, out-of-frame translation start site “Koz-ATG” flanked by two I-SceI sites 34 bp apart (Fig. 1a). Upon I-SceI expression, a DSB can be induced at either or both I-SceI sites. NHEJ repair of I-SceI-induced DSBs generates GFP^+^ cells because of pop-out of “Koz-ATG” by simultaneous DNA cleavage at two I-SceI sites, disruption of “Koz-ATG” by end resection initiated from either I-SceI-induced DSB, or correction of the GFP reading frame by indel-mediated loss of “3n + 1” bp or gain of “3n + 2” bp introduced at the downstream I-SceI cutting site. We previously analyzed a number of individual I-SceI-induced GFP^+^ clones by Sanger sequencing and a pool of sorted GFP^+^ cells by Illumina amplicon deep sequencing and showed that two distal and compatible DNA ends of I-SceI-induced DSBs were rejoined mostly by accurate NHEJ [11, 28].

Given that blunt ends of Cas9-induced DSBs could be directly ligated, we hypothesized that Cas9-induced DSBs also favor accurate NHEJ for their end-joining repair. As accurate NHEJ of a single DSB induced by Cas9 generates a product indistinguishable from uncut DNA, we thus designed paired Cas9-gRNAs, mimicking an I-SceI pair, to induce simultaneous Cas9 cleavage in the vicinity of two I-SceI sites in the reporter. We analyzed NHEJ by Illumina amplicon deep sequencing and compared accurate NHEJ repair of Cas9-induced DSBs with that of I-SceI-induced DSBs (Fig. 1a). The gRNA pair (i.e., gsGEJ2/gsGEJ2a), along with Cas9, induced DSBs at target sites that were 95 bp apart. For unbiased NHEJ analysis, we amplified the target DNA region of unsorted cells by PCR after NHEJ repair and analyzed repair junctions by Illumina deep sequencing. We categorized NHEJ repair of I-SceI- or Cas9-induced DSBs into four groups according to which two ends were ligated together (Fig. 1a): (1) group I for joining the distal ends of two DSBs induced by I-SceI or Cas9; (2) group II for rejoining two ends only at the first target site for I-SceI or Cas9; (3) group III for rejoining two ends only at the second target site for I-SceI or Cas9; (4) group IV for rejoining two ends respectively at each of two I-SceI or Cas9 target sites. In groups II–IV, products of accurate NHEJ were indistinguishable from the uncut templates and could not be counted. In group I, however, if distal ends were compatible or directly ligatable, accurate NHEJ occurred with deletion of the intervening DNA sequence between the two breakage sites and was therefore distinguishable and measurable. After normalization with transfection efficiencies, indicating the percentages of cells expressing I-SceI or Cas9, the level of Cas9-induced NHEJ was clearly higher than that of I-SceI-induced NHEJ (Fig. 1b). This is likely due to more efficient cleavage by Cas9. Group I events representing joining of distal ends of paired DSBs accounted for about 50% of I-SceI-induced NHEJ and over 65% of Cas9-induced NHEJ (Fig. 1c). While over 80% of group I events in I-SceI-induced NHEJ were accurate NHEJ, nearly 70% of Cas9-induced group I events were accurate (Fig. 1d). This suggested that Cas9-induced DSBs are more likely repaired by accurate NHEJ than mutagenic NHEJ.

To further test whether Cas9-induced DSBs are indeed repaired preferentially by accurate NHEJ, we designed paired gRNAs targeting an additional 70 genome sites in mouse and human cells and analyzed NHEJ repair of paired gRNA-guided Cas9-induced DSBs by deep sequencing (Additional file 1: Table S1). As the distance between two DSBs may have a suppressive effect on the accuracy and the efficiency of NHEJ [22, 29, 30], we restricted the distance between paired DSBs induced by Cas9 to less than 150 bp, with one exception of 1469 bp, to assess NHEJ of Cas9-induced DSBs, including accurate and mutagenic NHEJ. Among the 70 sites out of 71 analyzed, editing efficiencies varied significantly between 6.52% and 94.99% (mean ± standard deviation (SD), 35.87% ± 21.96%), group I between 1.82% and 96.01% (mean ± SD, 58.53% ± 20.80%), and accurate NHEJ between 0.00% and 94.05% (mean ± SD, 47.33% ± 27.59%) (Fig.1e). In particular, 49 out of 71 sites had accurate NHEJ over 30% of group I events and these accurate NHEJ products were also the most frequent single events in group I (Fig. 1e and Additional file 1: Table S1). Even among the 22 sites with a frequency lower than 30%, accurate NHEJ remained the most frequent single NHEJ events in four sites (Fig. 1e and Additional file 1: Table S1, S2). The difference was small in the efficiencies of accurate NHEJ between human cells and mouse ES cells (Additional file 2: Figure S1). Taken together, repair of Cas9-induced DSBs is inherently biased towards accurate NHEJ in both mouse and human cells with a frequency at about 50%.

We also performed three independent experiments to analyze NHEJ repair of paired DSBs induced by Cas9-gRNA on 4 genome sites, one at *PIF1* exon 1 of mouse ES cells (m*PIF1*), one at *LDHA* intron 5 of mouse ES cells (m*LDHA*), and two on *p53* exon 3 of human HEK293 cells (h*p53*#2 and h*p53*#3) (Additional file 1: Table S1). The editing efficiencies, i.e. the efficiencies of Cas9-induced NHEJ, were normalized with transfection efficiencies and varied from 14.41% to 81.80% among these 4 sites (Fig. 1f). These variations were likely due to the difference in either induction or repair of paired DSBs at these sites. Consistently, majority (71.70–95.81%) of Cas9-induced NHEJ was Group I events (Fig. 1g), in which accurate NHEJ accounted for 47.66%, 57.62%, 70.38% and 94.09% respectively (Fig. 1h). These data suggested that repair of Cas9-induced DSBs is inherently accurate.

### Accurate NHEJ is hindered by frequent + 1 templated insertions

**Fig. 2:**
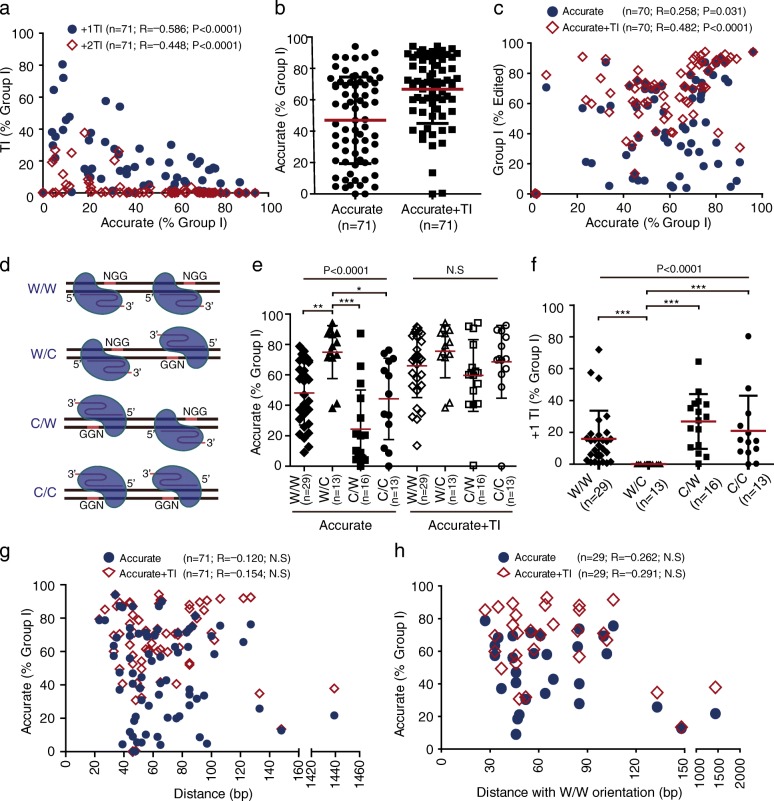
Accurate NHEJ is influenced by frequent + 1 templated insertions (*TI*), not directly by the orientations of paired Cas9 or the distance between target sites of paired Cas9. **a** Correlation between the frequency of accurate NHEJ and the frequency of + 1 TI (*blue dot*) or + 2 TI (*red rhombus*). *TI* template insertions with no additional mutations at repair junctions. **b** Comparisons between accurate NHEJ and accurate NHEJ combined with + 1 and + 2 template insertions (i.e., *Accurate+TI*). The difference indicates the underestimation of accurate NHEJ due to + 1 and + 2 TI. Student’s paired *t*-test *P* < 0.0001. **c** Correlation between the frequency of group I and the frequency of accurate NHEJ (*blue dot*) or the frequency of accurate NHEJ+TI (*red rhombus*). **d** Four different Cas9-gRNA orientations guided by paired PAMs. W/W, W/C, C/C, and C/W orientations were defined by the position of paired PAMs on either the Watson strand (W) or the Crick strand (C). **e** The frequency of Accurate or Accurate+TI from four orientations of 71 endogenous genome sites were calculated and summarized. Bars represent the mean ± standard deviation (SD). In the Accurate group, one-way ANOVA *P* < 0.0001; post LSD pairwise comparisons: **P < 0.01 for W/W vs W/C; ***P < 0.0005 for W/C vs C/W; *P < 0.05 for W/C vs C/C; not significant (NS) for the others. In the Accurate+TI group, one-way ANOVA NS. **f** The frequency of + 1 TI from four orientations of 71 endogenous genome sites were calculated and summarized. Bars represent the mean ± SD. One-way ANOVA *P* < 0.0001; post LSD pairwise comparison test ***P < 0.0005 for W/W vs W/C, ***P < 0.0005 for W/C vs C/W, ***P < 0.0005 for W/C vs C/C; not significant (*NS*) for the others. **g**, **h** Correlation between the frequency of accurate NHEJ (*blue circle*) or the frequency of Accurate+TI (*red rhombus*) and the distance between two cleavage sites at 71 genome sites (**g**) or 29 genome sites with the same W/W orientation (**h**)

Because the efficiency of accurate NHEJ at 22 out of 71 sites analyzed was less than 30% of group I events (Fig. 1e and Additional file 1: Table S1), we wondered why accurate NHEJ was inefficient in these cases. Examination of repair junctions revealed that + 1 insertions with no additional mutations were frequent and nearly all of them were templated, not random (Additional file 1: Table S3 and Additional file 2: Figure S2), confirming the findings of previous work [31, 32]. In fact, + 1 and + 2 templated insertions with no additional mutations occurred frequently (up to 85.83% of group I events) and were correlated inversely with accurate NHEJ (Fig. 2a). Over 10% of group I events were + 1 templated insertions in 36 out of 71 sites, among which + 2 templated insertions also accounted for over 10% of group I events in eight sites (Fig. 2a and Additional file 1: Table S3). The + 1 or + 2 templated insertions had frequencies over 30% of group I even at 11 sites and were the most frequent at 14 sites (Additional file 1: Tables S2 and S3).

Previous work has proposed that Cas9 cleavage could generate not only blunt ends but also 1- or 2-nucleotide (nt) 5′-overhanging ends because the RuvC nuclease domain of Cas9 could cleave at the fourth or fifth base upstream of a PAM in addition to the third [3, 31–33]. These 5′-overhanging ends are filled in by DNA polymerases, thus producing + 1 or + 2 templated insertions [31]. The observed high levels of + 1 or + 2 templated insertions indicate that Cas9 cleavage can generate 1- or 2-nt 5′-overhanging ends frequently. However, at individual breakage sites of paired DSBs, the 1- or 2-nt 5′-overhanging ends are complementary and, like blunt ends, can be rejoined readily and accurately, thus preventing templated insertions. In contrast, distal ends of paired DSBs with 1- or 2-nt 5′-overhangs may be incompatible for accurate end joining, and filling in these ends by DNA polymerases can thus generate templated insertions in group I. Consequently, the frequency of accurate end joining in group I may underestimate the true frequency of accurate NHEJ in repair of Cas9-induced DSBs. To better reflect accurate NHEJ of Cas9-induced DSBs, we combined accurate end joining in group I with templated insertion events that had no additional indels and termed them “Accurate+TI”. Compared to the frequency of accurate NHEJ—49.74% on average—the average frequency of “Accurate+TI” was 66.88%, indicating that accurate NHEJ of Cas9-induced DSBs might be underestimated by 17.14% (Fig. 2b). Because accurate rejoining of blunt ends or complementary 5′-overhanging ends at individual breakage sites provides templates for recleavage by Cas9, we speculated that this accurate repair would increase the simultaneous cleavage by paired gRNA-guided Cas9, promoting group I NHEJ. Indeed, the frequency of group I events was positively correlated with that of Accurate+TI (Fig. 2c).

The stable binding of Cas9 to target DNA is directed by the position of the PAM (i.e., NGG) on either the Watson strand (W) or the Crick strand (C). The orientation of paired PAMs for paired Cas9-gRNAs has four different combinations: W/W, W/C, C/W and C/C (Fig. 2d). We wondered whether the difference in paired PAM orientations would affect the efficiency of accurate NHEJ. As revealed, while the difference was not significant in the efficiency of accurate NHEJ between W/W, C/C, and C/W, accurate NHEJ induced by W/C was more efficient than that by W/W, C/C, and C/W (one-way ANOVA, *P* < 0.0001; post hoc pairwise comparison, *P* < 0.05; Fig. 2e). However, when accurate NHEJ was combined with templated insertions (i.e., “Accurate+TI”), the difference induced by W/C was lost (Fig. 2e). This implied that W/C and the other three combinations of PAM orientations might induce different frequencies of templated insertions. In fact, W/C generated few templated insertions whereas W/W, C/W, and C/C gave rise to frequent + 1 or + 2 templated insertions at many sites (one-way ANOVA, *P* < 0.0001; post hoc pairwise comparison, *P* < 0.005 for W/C vs others and *P* > 0.05 for other comparisons; Fig. 2f). This could be predicted according to the position of simultaneous Cas9 cleavage that deletes the intervening sequence and generates 1-nt 5′-overhanging ends (Additional file 2: Figure S3). Nearly all of + 1 templated insertions were as predicted (Additional file 2: Figure S4). This suggested that the W/C orientation, not W/W, C/W, or C/C, could avoid the interference of + 1 and + 2 templated insertions on accurate NHEJ.

To determine whether the distance between paired DSBs induced by Cas9 affects the efficiency of accurate NHEJ, we performed correlation analysis on the data from all 71 sites. The frequencies of accurate NHEJ or Accurate+TI were not correlated with the distance between paired DSBs (Fig. 2g). Given that 70 sites out of 71 were packed within the range 23–148 bp, this indicated that this distance has no effect on accurate NHEJ whether or not accurate NHEJ is combined with templated insertions. To exclude the possible interference of the PAM orientations in the analysis, we assessed the data only with the W/W orientation of the PAMs. Consistently, we did not find any significant effect of the distance on accurate NHEJ or Accurate+TI (Fig. 2h). Therefore, it appears that accurate NHEJ is not affected by the distance between paired DSBs within the range 23–148 bp.

### Validation of a reporterless NHEJ assay in cells and in vivo

**Fig. 3:**
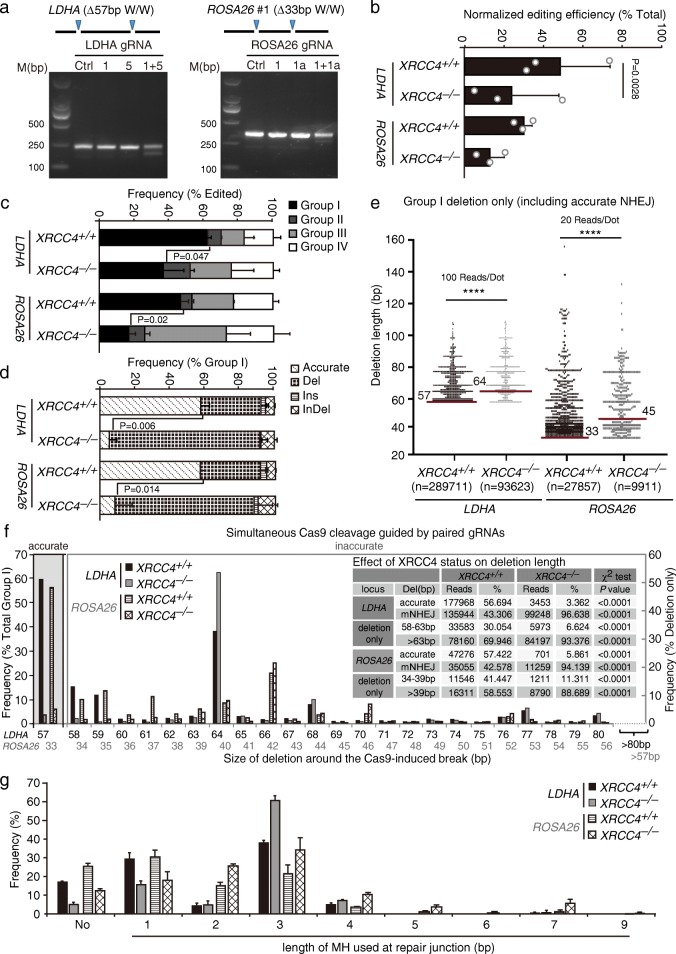
Validation of a reporter-less NHEJ assay in cells deficient for *XRCC4*. **a** Deletion of the intervening sequence between two target sites by paired Cas9-gRNA. Cells were transfected with expression plasmids for single or paired sgRNAs and Cas9 and genomic DNA was purified 72 h post-transfection and amplified by primers flanking the cutting sites. The PCR amplicons were subjected to agarose gel electrophoresis. The distance between two cleavage sites was 57 bp at the *LDHA* site and 33 bp at the *ROSA26* site as indicated and the deletion of the intervening sequence indicates simultaneous Cas9 cleavage. **b–d** Analysis of NHEJ induced by paired Cas9-gRNA at the *LDHA* and *ROSA26* sites in isogenic *XRCC4*^*+/+*^ and *XRCC4*^*−/−*^ mouse ES cells. The normalized editing efficiency (**b**), the frequency of each group in edited events (**c**), and the frequency of each category in group I events (**d**) were calculated. The normalized editing efficiency represents the efficiency of overall NHEJ, including accurate and mutagenic NHEJ. Bars represent the mean ± standard deviation (SD) of three independent experiments. For normalized editing efficiency (**b**), Student’s paired *t*-test between *XRCC4*^*+/+*^ and *XRCC4*^*−/−*^ *P* = 0.0028 at the *LDHA* site. **e** Deletion length distributions of Group I ‘Del’ events in isogenic *XRCC4*^*+/+*^ and *XRCC4*^*−/−*^ mouse ES cells. The reads were combined by three independent experiments. At the *LDHA* site and the *ROSA26* site, each *dot* represents 100 reads and 20 reads, respectively. The median deletion length is indicated, and deletion distributions demonstrate a shift towards longer deletions in cells lacking *XRCC4* (Mann–Whitney test, ****P < 0.0001). **f** Frequency of accurate NHEJ among group I (*left*) and frequency of deletions with different deletion length in “Del” events of group I NHEJ (*right*) in either isogenic *XRCC4*^*+/+*^ or *XRCC4*^*−/−*^ mouse ES cells. Del NHEJ events were grouped into 58–63 bp and > 63 bp at the *LDHA* site and 34–39 bp and > 39 bp at the *ROSA26* site. The respective reads and frequencies were summarized in the inset and compared by a χ^2^ test with *P* values indicated. **g** Frequency of microhomology usage at different lengths in deletion-only group I events of either isogenic *XRCC4*^*+/+*^ or *XRCC4*^*−/−*^ mouse ES cells

As described above, in the repair of two adjacent DSBs induced simultaneously by paired Cas9-gRNAs, accurate NHEJ and mutagenic NHEJ could be distinguished and measured. This provides a convenient method to analyze NHEJ, including both accurate and mutagenic NHEJ in cells and in vivo, without using an NHEJ reporter. To further validate this approach, we selected the *LDHA* locus and the *ROSA26* locus (m*ROSA26*#1) that were analyzed before in mouse ES cells (Additional file 1: Table S1) and examined the effect of *XRCC4* loss on Cas9-induced NHEJ. As a core NHEJ factor for end ligation during NHEJ, XRCC4 may also control end ligation for Cas9-induced NHEJ. We thus determined whether *XRCC4* regulates Cas9-induced NHEJ in the same way as in our previous NHEJ reporter assay with I-SceI [11]. Due to precise deletions of the 57-bp or 33-bp intervening sequence between paired Cas9-gRNA target sites at the *LDHA* and *ROSA26* loci, respectively, two major bands were separated by DNA gel electrophoresis for PCR products of the target DNA after repair (Fig. 3a). The upper band was expected to contain the unedited and edited targets with small indels and the lower band most likely the edited target with deletion of the intervening sequence. The intensity of the lower band relative to the upper band reflected the efficiency of simultaneous cleavage by paired Cas9-gRNAs, providing a rapid and convenient method to identify efficient paired gRNAs (Fig. 3a).

After induction of paired DSBs on the *LDHA* site or the *ROSA26* site in isogenic *XRCC4*^*+/+*^ and *XRCC4*^*−/−*^ mouse ES cells and subsequent repair, we performed Illumina amplicon sequencing of repair junctions. Examination of repair junctions revealed that the overall editing efficiency decreased from 47.70 ± 25.10% in *XRCC4*^*+/+*^ cells to 23.57 ± 23.93% in *XRCC4*^*−/−*^ cells at the *LDHA* site and from 29.50 ± 4.15% in *XRCC4*^*+/+*^ cells to 12.56 ± 7.18% in *XRCC4*^*−/−*^ cells at the *ROSA26* site (Fig. 3b). Given the difference in the expression levels of gRNA and Cas9 and the Cas9 cleavage efficiency between *XRCC4*^*+/+*^ and *XRCC4*^*−/−*^ cells and between experiments, the editing efficiencies were normalized with the transfection efficiencies of either cells in each independent experiment as described previously [28]. As the normalized editing efficiency reflects the efficiency of overall NHEJ, its reduction in *XRCC4*^*−/−*^ cells suggested that *XRCC4* facilitates NHEJ in repair of Cas9-induced DSBs as in repair of I-SceI-induced DSBs. In edited products, group I events were also reduced from 62.06 ± 2.34% in *XRCC4*^*+/+*^ cells to 36.68 ± 11.81% in *XRCC4*^*−/−*^ cells at the *LDHA* site and from 46.70 ± 4.53% in *XRCC4*^*+/+*^ cells to 16.47 ± 3.92% in *XRCC4*^*−/−*^ cells at the *ROSA26* site (Fig. 3c). This indicated that *XRCC4* biases NHEJ towards group I. Accurate NHEJ of Cas9-induced DSBs was also dramatically reduced from 57.62 ± 4.88% in *XRCC4*^*+/+*^ cells to 5.47 ± 4.23% in *XRCC4*^*−/−*^ cells at the *LDHA* site and from 57.12 ± 3.20% in *XRCC4*^*+/+*^ cells to 8.62 ± 9.72% in *XRCC4*^*−/−*^ cells at the *ROSA26* site (Fig. 3d). This is in line with previous observations that *XRCC4* promotes accurate NHEJ of I-SceI-induced DSBs [11, 15].

Furthermore, compared to *XRCC4*^*+/+*^ cells, *XRCC4*^*−/−*^ cells were biased towards longer deletion length (Fig. 3e; Mann-Whitney test, *P* < 0.0001). The median length of group I deletion in *XRCC4*^*−/−*^ cells was 64 bp (i.e., 7 bp + 57-bp pop-out) at the *LDHA* site and 45 bp (i.e., 12 bp + 33-bp pop-out) at the *ROSA26* site, longer, respectively, than 57 bp and 33 bp in *XRCC4*^*+/+*^ cells (Fig. 3e). The *XRCC4* deficiency caused more frequent mutagenic NHEJ events with deletions of over 63 bp (i.e., 6 bp + 57-bp pop-out) at the *LDHA* site (93.38% vs 69.95% between *XRCC4*^*−/−*^ and *XRCC4*^*+/+*^ cells; χ^2^ test, *P* < 0.0001) and over 39 bp (i.e., 6 bp + 33-bp pop-out) at the *ROSA26* site (88.69% vs 58.55% between *XRCC4*^*−/−*^ and *XRCC4*^*+/+*^ cells; χ^2^ test, *P* < 0.0001), but less with deletions of 58–63 bp (i.e., 1–6 bp + 57-bp pop-out) at the *LDHA* site (6.62% vs 30.05% between *XRCC4*^*−/−*^ and *XRCC4*^*+/+*^ cells; χ^2^ test, *P* < 0.0001) and 34–39 bp at the *ROSA26* site (i.e., 1–6 bp + 33-bp pop-out) (11.31% vs 41.45% between *XRCC4*^*−/−*^ and *XRCC4*^*+/+*^ cells; χ^2^ test, *P* < 0.0001) (Fig. 3f and inset). These data demonstrate that *XRCC4* promotes accurate NHEJ and suppresses mutagenic NHEJ in repair of Cas9-induced DSBs.

Previous studies have shown that loss of *XRCC4* promotes increased use of microhomologies (MH) in NHEJ [11, 15, 34]. We thus examined the use of MH in Cas9-induced NHEJ and observed that the frequency of NHEJ with no MH at repair junctions was much higher in *XRCC4*^*+/+*^ cells than in *XRCC4*^*−/−*^ cells (Fig. 3g), indicating increased use of MH in Cas9-induced NHEJ in the absence of *XRCC4*. Especially, the use of 3-bp MH increased from 40.50% and 21.80% in *XRCC4*^*+/+*^ cells to 64.95% and 31.30% in *XRCC4*^*−/−*^ cells at the *LDHA* and *ROSA26* sites, respectively (Fig. 3g), whereas the use of 1-bp MH decreased from 31.25% and 31.17% in *XRCC4*^*+/+*^ cells to 16.68% and 16.32% in *XRCC4*^*−/−*^ cells. Notably, due to the presence of 3-bp MH (i.e., GTG at the *LDHA* site and CAC at the *ROSA26* site), the frequencies of 64-bp deletions (i.e., 7 bp + 57-bp pop-out) at the *LDHA* site and 42-bp deletions (i.e., 9 bp + 33-bp pop-out) at the *ROSA26* site were quite high in *XRCC4*^*+/+*^ cells and were further increased in *XRCC4*^*−/−*^ cells (Fig. 3g).

**Fig. 4:**
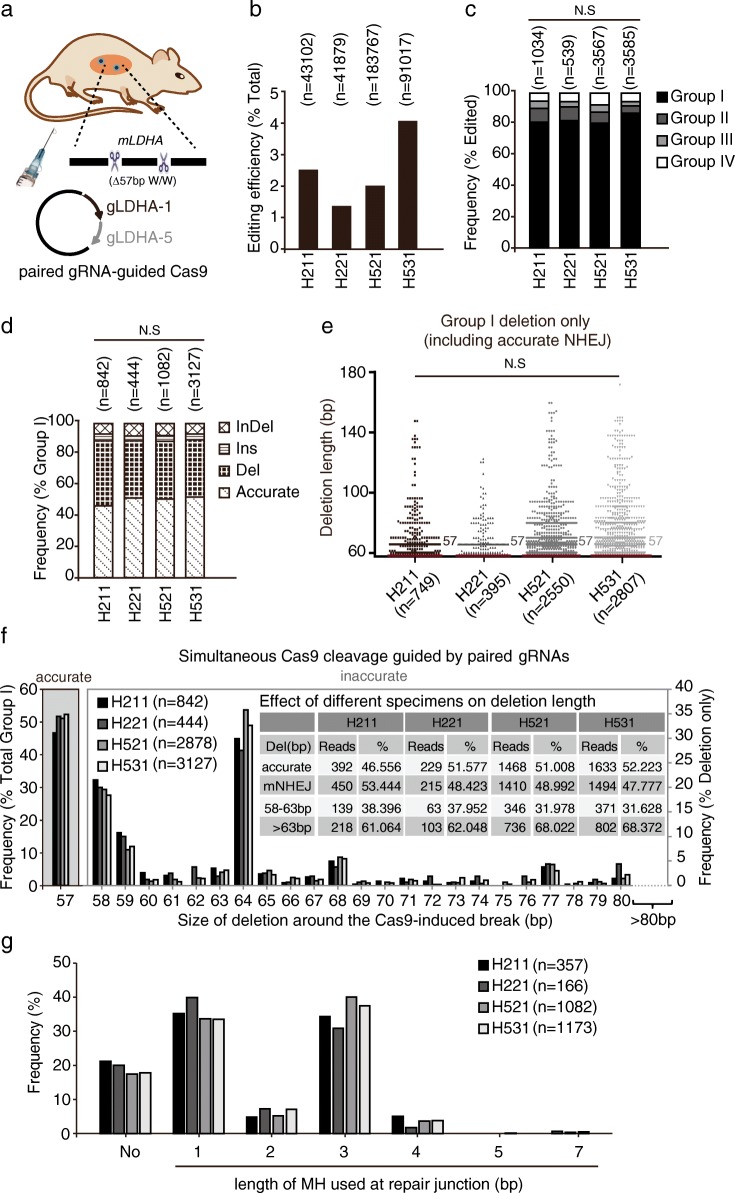
Validation of a reporterless NHEJ assay in mouse liver. **a** Delivery of paired Cas9-gRNAs into mouse liver by hydrodynamic tail vein injection. **b**–**d** Analysis of NHEJ induced by paired Cas9-gRNA at the LDHA sites in mouse liver. The editing efficiency (**b**), the frequency of each group in edited events (**c**), and the frequency of each category in group I events (**d**) were calculated (Mann–Whitney test, not significant (*NS*)). **e** Deletion length distributions of group I “Del” events in mouse livers. The median deletion length is indicated, and deletion length distributions demonstrate no shift towards longer deletions between these liver specimens (Kruskal–Wallis test, NS, *P* = 0.1922). **f** Frequency of accurate NHEJ among group I (*left*) and frequency of deletions with different deletion lengths in Del events of group I NHEJ (*right*) in mouse livers. Del NHEJ events were grouped into 58–63 bp and > 63 bp at the *LDHA* site. The respective reads and frequencies are summarized in the inset. **g** Frequency of the microhomology usage at different lengths in deletion-only group I events of mouse liver

To test whether this reporterless NHEJ assay can be applied in vivo, we delivered the expression plasmids for paired Cas9-gRNAs targeting the *LDHA* site into two mice through hydrodynamic tail vein injection, harvested livers 30 days post-injection, and analyzed accurate and mutagenic NHEJ (Fig. 4a). In four pieces of mouse liver tissue, two each mouse, the editing efficiencies ranged from 1 to 4%, much lower than that in cultured cells due to inefficient delivery of plasmids into mouse liver (Fig. 4b). More than 80% of the edited events were group I NHEJ products (Fig. 4c) and approximately a half of group I were accurate NHEJ (Fig. 4d). This indicated that accurate NHEJ is also innately favored in mouse liver in repair of Cas9-induced DSBs. Further analysis of group I events revealed little difference in the deletion pattern (Fig. 4e, f) and in the MH usage in Cas9-induced NHEJ among four liver samples (Fig. 4g). Moreover, the frequency of accurate NHEJ, the deletion pattern, and the MH usage in mouse liver were similar to those in cultured mouse ES cells (Fig. 4d–g). Therefore, the reporterless NHEJ assay described here could be used as a convenient, flexible, and accurate method to analyze both accurate and mutagenic NHEJ in cells and in vivo.

### Precise out-of-frame deletion mediated by accurate NHEJ improves gene knockout

**Fig. 5:**
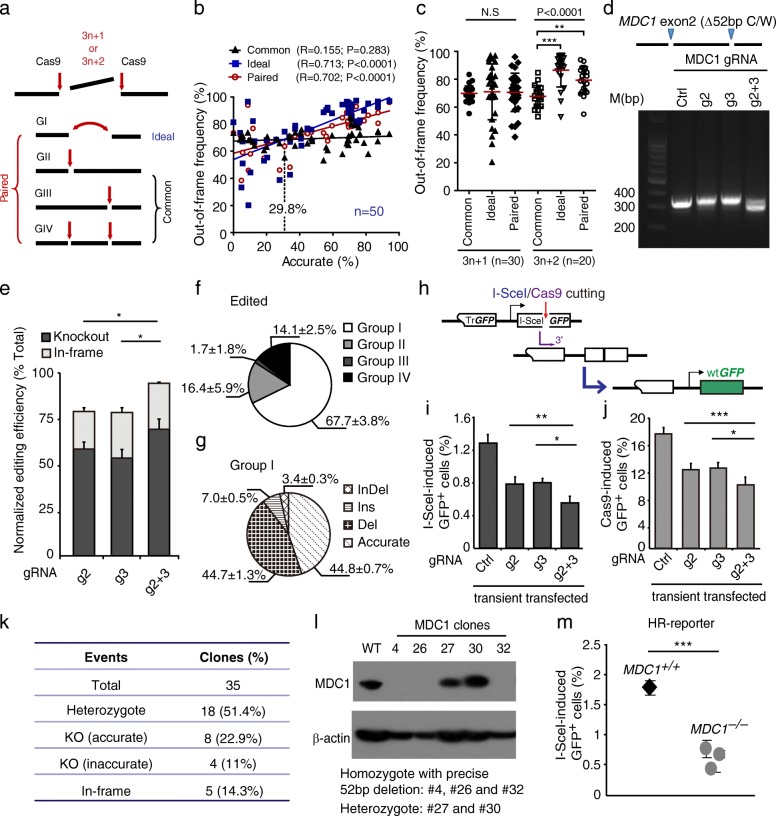
Precise out-of-frame deletion mediated by accurate NHEJ promotes gene knockout. **a** Classification of the Common, Ideal, and Paired approaches. With paired Cas9-gRNAs, Common, Ideal, and Paired represent genome editing that depends, respectively, upon NHEJ without group I events, NHEJ for ideally all simultaneous Cas9 cleavage, and NHEJ including group I events. **b** Correlation between accurate NHEJ and out-of-frame mutations derived from the Common approach (*black squares*), the Ideal approach (*blue squares*), or the Paired approach (*red circle*). **c** The frequency of out-of-frame mutations between different groups as indicated. Bars represent the mean ± standard deviation (SD) of the out-of-frame frequencies. Data were analyzed by one-way ANNOVA followed by post-hoc LSD pairwise comparisons (**P < 0.005, ***P ≤ 0.0005, *NS* not significant). **d** Testing of paired Cas9-gRNA at exon 2 of *mMDC1*. Mouse ES cells were transfected with expression plasmids for single or paired gRNAs and Cas9 and genomic DNA were purified 72 h post-transfection and amplified by primers flanking the cutting sites. The PCR amplicons were subjected to agarose gel electrophoresis. The distance between paired Cas9-gRNA target sites is 52 bp as indicated. **e** The frequency of out-of-frame (i.e., knockouts) and in-frame *MDC1* editing events induced by single or paired Cas9-gRNA. For knockout efficiency, Student’s paired *t*-test between g2 and g2 + 3 *P = 0.029; between g3 and g2 + 3 *P = 0.013. **f** The frequency of group I, group II, group III, and group IV events among those edited by Cas9 guided by paired gRNAs as indicated. **g** The frequency of accurate, deletion, insertion, and indel events among group I events induced by paired Cas9-gRNAs. **h** The HR reporter. Repair of an I-SceI- or Cas9-induced DSB by HR between sister chromatids generates wild-type *GFP*. **i**, **j** Percentage of I-SceI- (**i**) or Cas9- (**j**) induced GFP^+^ cells from HR reporter mouse ES cells transiently transfected with expression plasmids for single or paired gRNA-guided Cas9. Values are the mean ± SD of three independent experiments, each in triplicate. In I-SceI-induced HR (**i**), Student’s paired *t*-test between g2 and g2 + 3 **P = 0.0048; between g3 and g2 + 3 *P = 0.0154. In Cas9-induced HR assays (**j**), Student’s paired *t*-test between g2 and g2 + 3 ***P = 0.0004; between g3 and g2 + 3 *P = 0.0449. **k** Generation of *MDC1* mutant clones by paired Cas9-gRNAs. Clones were picked, grown, and identified by Sanger sequencing. The frequencies of specific *MDC1* mutations are indicated in parentheses. **l** *MDC1* knockout was confirmed by western blotting using β-actin as a loading control. **m** Percentage of I-SceI-induced GFP^+^ cells from one *MDC1*^*+/+*^ and three isogenic *MDC1*^*−/−*^ HR reporter clones (clones #4, #26, and #32). Bars represent the mean ± SD of three independent experiments, each in triplicate. Student’s unpaired *t*-test: ***P < 0.0001 between *MDC1*^*+/+*^ and *MDC1*^*−/−*^. *WT* wild type

Among major applications of CRISPR/Cas9 genome editing, gene knockouts are originated mostly from heterogeneous out-of-frame indels induced during NHEJ repair of Cas9-induced DSBs [1, 2]. Given efficient generation of precise deletions of defined length by accurate NHEJ via paired Cas9-gRNAs, we wondered whether accurate NHEJ could be harnessed to improve the efficiency and homogeneity of gene knockouts. Thus, we first compared the efficiency of out-of-frame indels induced by this paired Cas9-gRNA method involving accurate NHEJ (termed “Paired”) with the common dual gRNA-guided editing approach (termed “Common”), which is represented by the paired Cas9-gRNA method excluding group I NHEJ. Group I NHEJ alone mimicked an ideal paired Cas9-gRNA method (termed “Ideal”), where all of Cas9 cleavage guided by paired gRNAs is concurrent, thereby editing the genome all by group I NHEJ (Fig. 5a). Analysis revealed that the frequency of out-of-frame mutations induced by the Paired approach and by the Ideal approach, but not by the Common approach, was positively correlated with the frequency of accurate NHEJ (Fig. 5b). With accurate NHEJ at a frequency over 29.8%, both the Paired and the Ideal approaches generated out-of-frame mutations more efficiently than the Common method, and the more efficient accurate NHEJ was, the better improvement in the efficiency of out-of-frame mutations (Fig. 5b and Additional file 2: Figure S7). For example, with the frequency at 68.36%, accurate NHEJ increased the knockout efficiency from 65.52% to 88.51% at a site of the mouse *Artemis* locus (Fig. 5b and Additional file 1: Table S4). By contrast, the out-of-frame editing efficiency tended to be reduced by accurate NHEJ at a level below 30% (Fig. 5b).

Due to the inverse relationship between accurate NHEJ and frequent + 1 templated insertions, we speculated that frequent + 1 templated insertions might reduce the out-of-frame editing frequency. In fact, the frequencies of + 1 templated insertions varied inversely with those of out-of-frame mutations (Additional file 2: Figure S8). Importantly, with deletions of predefined 3n + 1 bp, the frequencies of + 1 templated insertions were strongly and negatively correlated with the frequencies of out-of-frame mutations induced by Ideal and by Paired methods (Additional file 2: Figure S6). When the frequency of + 1 templated insertions was less than 20.5%, accurate NHEJ resulting in precise deletions of defined 3n + 1-bp length was able to improve the out-of-frame frequency, and the less the frequency of + 1 templated insertions, the better the improvement (Additional file 2: Figure S6). In contrast, no correlation was observed between + 1 templated insertions and deletions of predefined 3n + 2 bp or out-of-frame mutations induced by the Common approach (Additional file 2: Figure S6). This indicated that + 1 templated insertions might convert out-of-frame deletions of 3n + 1 bp, but not 3n + 2 bp, into in-frame deletions of 3n bp, thus reducing the out-of-frame efficiency. As a result, the out-of-frame editing efficiency was improved by deletions of predefined 3n + 2 bp via accurate NHEJ, but not by 3n + 1 bp, in both Ideal and Paired approaches when + 1 templated insertions were frequent (Fig. 5c). Among 20 genome sites edited with deletions of predefined 3n + 2 bp, the out-of-frame efficiency was increased, on average, from 67.76% to 86.48% in the Ideal method (Student’s paired *t*-test, *P* < 0.0001) and from 67.76% to 79.26% in the Paired approach (Student’s paired *t*-test, *P* < 0.0001) (Fig. 5c). This suggested that the distance between paired Cas9-gRNA cleavage sites should be preset preferably at 3n + 2 bp rather than 3n + 1 bp in order to improve the efficiency of out-of-frame mutations, including gene knockouts. Only when the frequency of templated insertions is low could 3n + 1 bp be useful.

To validate the application of accurate NHEJ induced by paired Cas9-gRNAs in out-of-frame mutations, we knocked out the *MDC1* gene in mouse ES cells by targeting exon 2 of *MDC1* (m*MDC1*#2) with paired Cas9-gRNA as an example (Fig. 5d and Additional file 1: Table S1). Between two cleavage sites was a 52-bp intervening sequence. We transfected cells with the expression plasmids for single gRNA or paired gRNAs, along with Cas9, isolated genomic DNA at 72 h post-transfection, and amplified the target sequence by PCR. Compared to single gRNA or the empty vector control, transfection with paired gRNAs g2 and g3 generated a weaker 350-bp upper band and a much stronger 300-bp lower band (Fig. 5d), indicating that these two gRNAs were not only individually effective but also highly efficient in inducing concurrent Cas9 cleavage. Further analysis of the repair junctions revealed that the editing efficiencies of single gRNAs and paired gRNAs were about 80% and 96.20%, respectively, after normalization with transfection efficiencies (Fig. 5e). Among these editing efficiencies, the knockout frequency was higher with paired gRNAs at 70.4% than single gRNAs at 50–60% (Fig. 5e). In the edited events, 67.74% were group I events, confirming that concurrent Cas9 cleavage guided by paired gRNAs was highly efficient (Fig. 5f). Notably, accurate NHEJ accounted for 44.83% of group I events (Fig. 5g).

Because paired Cas9-gRNA induced a higher frequency of *MDC1* knockouts, we predicted that paired gRNAs should be more effective in disrupting the function of *MDC1* than single gRNA. We thus performed homologous recombination (HR) assays with *MDC1* gRNA in HR reporter mouse ES cells previously established [13]. In this HR reporter, the frequency of I-SceI- or Cas9-induced GFP^+^ cells reflects the relative efficiency of HR (Fig. 5h). We found that single gRNA reduced I-SceI- or Cas9-induced HR by about 25% and paired gRNAs by nearly a half compared to the empty vector negative control (Fig. 5i, j). This indicated that paired gRNAs are more effective in inactivating *MDC1* than single gRNA.

Next, we applied paired gRNAs to generate *MDC1* knockout clones. After transfection of mouse HR reporter ES cells with the expression plasmids for paired gRNAs and Cas9, these cells were sparsely plated and grown without any selection. In 2 weeks, we randomly picked 35 clones and examined by PCR and Sanger sequencing the target sites. *MDC1* was edited in all these 35 clones; 18 were heterozygotes and 17 homozygotes (Fig. 5k). Among these homozygotes, eight contained precise 52-bp deletions mediated by accurate NHEJ in both alleles and were therefore knockout clones (Fig. 5k). Of the remaining nine clones, four were knockout clones carrying additional mutations besides deletion of the 52-bp intervening sequence, and five had in-frame mutations (Fig. 5k). It was apparent that the heterogeneity of indels was low in *MDC1* editing by paired Cas9-gRNAs. *MDC1* knockout was further confirmed by western blotting with anti-MDC1 antibody (Fig. 5l). We performed HR assays in three *MDC1* knockout clones that have a precise 52-bp deletion on both alleles and parental *MDC1* wild-type cells. These *MDC1* knockout clones have a lower level of I-SceI-induced HR, approximately 0.5%, compared to parental cells at 1.8% (Fig. 5m). These results demonstrated that accurate NHEJ in the paired Cas9-gRNA method can improve the efficiency and reduce the heterogeneity of gene knockouts with precise deletions of defined length in CRISPR/Cas9 genome editing.

### Targeted in-frame deletion mediated by accurate NHEJ assists functional domain analysis in situ

**Fig. 6:**
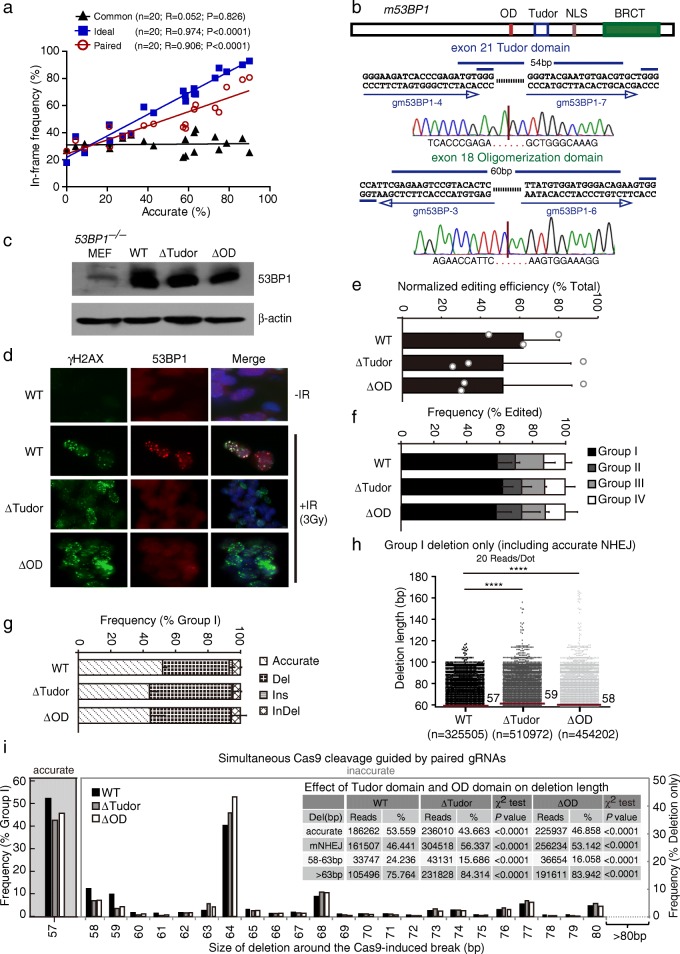
Targeted in-frame deletions mediated by accurate NHEJ assists functional domain analysis in situ. **a** Correlation between accurate NHEJ and in-frame deletions generated by the Common (*black triangles*), Ideal (*blue squares*), and Paired approaches (*red circles*). **b** Domain structure of mouse 53BP1 protein. Oligomerization domain (*OD*), Tudor domain, nuclear localization sequence (*NLS*), and tandem BRCT domain are shown. Two sets of paired gRNAs respectively targeting the Tudor domain (exon 21) and OD domain (exon 18) are also indicated. Precise in-frame deletions were confirmed by Sanger sequencing of the PCR product for repair junction. **c** Detection of *53BP1* wild-type and in-frame mutants by western blotting using β-actin as loading control. *WT* wild type. **d** IR-induced foci formation of γH2AX (*red*) and 53BP1 (*green*) in *53BP1* WT clone and in-frame mutant clones. **e**–**g** Analysis of NHEJ induced by paired Cas9-gRNAs at the *LDHA* site in *53BP1* WT clone and in-frame mutant clones. The normalized editing efficiency (**e**), the frequency of each group in edited events (**f**), and the frequency of each category in group I events (**g**) were calculated. Bars represent the mean ± standard deviation (SD) of three independent experiments. **h** Deletion length distributions of group I “Del” events at the LDHA site in *53BP1* WT mouse ES clone and in-frame mutant clones. The reads were combined from three independent experiments. Each *dot* represents 20 reads. The median deletion length is indicated, and deletion length distributions demonstrate a shift towards longer deletions in *53BP1* in-frame mutant clones (Mann–Whitney test between WT and ΔTudor or ΔOD ****P < 0.0001). **i** Frequency of accurate NHEJ among group I (*left*) and frequency of deletions with different deletion length in Del events of group I NHEJ (*right*) in *53BP1* WT mouse ES clone and in-frame mutant clones. Del NHEJ events were grouped into 58–63 bp and > 63 bp. The respective reads and frequencies are summarized in the inset and compared by a χ^2^ test with *P* values as indicated

To evaluate whether paired Cas9-gRNAs can generate efficient precise in-frame deletion mediated by accurate NHEJ, we first examined the repair junction data from 20 genome sites and determined whether accurate NHEJ could indeed increase in-frame mutations induced by the Ideal or the Paired method (Additional file 1: Table S4). The data showed that the frequency of in-frame indels was strongly and positively correlated with the frequency of accurate NHEJ that generated precise deletions of 3n bp (Fig. 6a and Additional file 1: Table S4). Compared to the Common approach, the frequency of in-frame indels was increased from 31.43% to 59.88% on average by the Ideal method (Student’s paired *t*-test, *P* < 0.0001) and from 31.43% to 49.29% by the Paired method (Student’s paired *t*-test, *P* < 0.001) (Additional file 2: Figure S9). This suggests that the more efficient accurate NHEJ is, the better the improvement in the in-frame editing efficiency.

To further validate the application in targeted in-frame deletions, we designed two sets of paired gRNAs to target exon 18 and exon 21 of the DNA damage response gene *53BP1* for precise in-frame deletions mediated by accurate NHEJ. We generated two *53BP1* mutant clones, which were confirmed by PCR and Sanger sequencing. One had precise biallelic deletion of the predefined 60-bp intervening sequence in the oligomerization domain (ΔOD) and the other precise biallelic deletion of the predefined 54-bp intervening sequence in the Tudor domain (ΔTudor) (Fig. 6b). These two small, in-frame deletions do not affect the size and the steady-state level of truncated 53BP1 proteins (Fig. 6c), suggesting that truncated 53BP1 is fully translated and stable. However, when we treated mouse ES cells with ionized radiation (IR) of 3 Gy, no 53BP1 foci were detected in the *53BP1* ΔTudor and ΔOD clones, while wild-type 53BP1 formed IR-induced nuclear foci (Fig. 6d). This is consistent with previous findings that both OD and Tudor domains are important for IR-induced 53BP1 focus formation [35–37]. IR-induced γH2AX foci were normally formed in both the 53BP1 wild-type clone and mutant clones as expected (Fig. 6d).

53BP1 is thought to protect DSBs from end resection in G1 phase of the cell cycle and antagonize BRCA1 in S and G2 [37, 38]. We reasoned that the inability of 53BP1ΔTudor and 53BP1ΔOD to bind the damaged chromatin would limit the end-protection function of *53BP1*, thus increasing end resection of DSBs, including Cas9-induced DSBs, and affecting the efficiency and the accuracy of NHEJ. Thus, using our reporterless NHEJ assay described above, we analyzed repair junctions of the *LDHA* target site in the *53BP1*ΔTudor clone and the *53BP1*ΔOD clone as well as *53BP1* wild-type mouse ES cells. Disruption of either Tudor or OD had little effect on the normalized editing efficiency (Fig. 6e) and the frequency of group I events (Fig. 6f). There was a small but insignificant difference in the frequency of accurate NHEJ between wild-type cells and the *53BP1*ΔTudor clone or the *53BP1*ΔOD clone (Fig. 6g). However, deletions were shifted towards modestly longer length in 53BP1ΔTudor cells and in 53BP1 ΔOD cells (Fig. 6h; Mann-Whitney test, *P* < 0.0001). The median length of group I deletion was 59 bp in *53BP1*ΔTudor cells and 58 bp in *53BP1*ΔOD cells, 1–2 bp longer than 57 bp in *53BP1*^*+/+*^ cells (i.e., 57-bp pop-out; Fig. 6h).

Further examination of the combined data from three independent experiments revealed that the level of accurate NHEJ was lower in both *53BP1*ΔTudor and *53BP1*ΔOD cells than in wild-type parental cells (Fig. 6i and inset; χ^2^ test, *P* < 0.0001). Both *53BP1*ΔTudor and *53BP1*ΔOD cells generated less mutagenic NHEJ events with deletions of 58–63 bp (i.e., 1–6 bp + 57-bp pop-out), 15.69% and 16.06%, respectively, vs 24.24% in *53BP1* wild-type cells (Fig. 6i and inset; χ^2^ test, *P* < 0.0001). In contrast, deletions of over 63-bp (i.e., 6 bp + 57-bp pop-out) were more frequent in *53BP1*ΔTudor cells at 84.31% and in *53BP1*ΔOD cells at 83.94% than *53BP1* wild-type cells at 75.76% (Fig. 6i and inset; χ^2^ test, *P* < 0.0001). However, the MH usage in mutagenic NHEJ was not significantly altered between *53BP1* wild-type cells and *53BP1* mutant cells (Additional file 2: Figure S10). This suggested that 53BP1 may suppress end resection at close ends of Cas9-induced DSBs by locating to the damaged chromatin, thus biasing NHEJ away from longer deletions. These results also demonstrate that targeted in-frame deletions mediated by accurate NHEJ can be harnessed to efficiently and accurately disrupt a particular motif or domain of a protein for functional analysis.

### The Plk3 inhibitor GW843682X enhances accurate NHEJ in repair of Cas9-induced DSBs

**Fig. 7:**
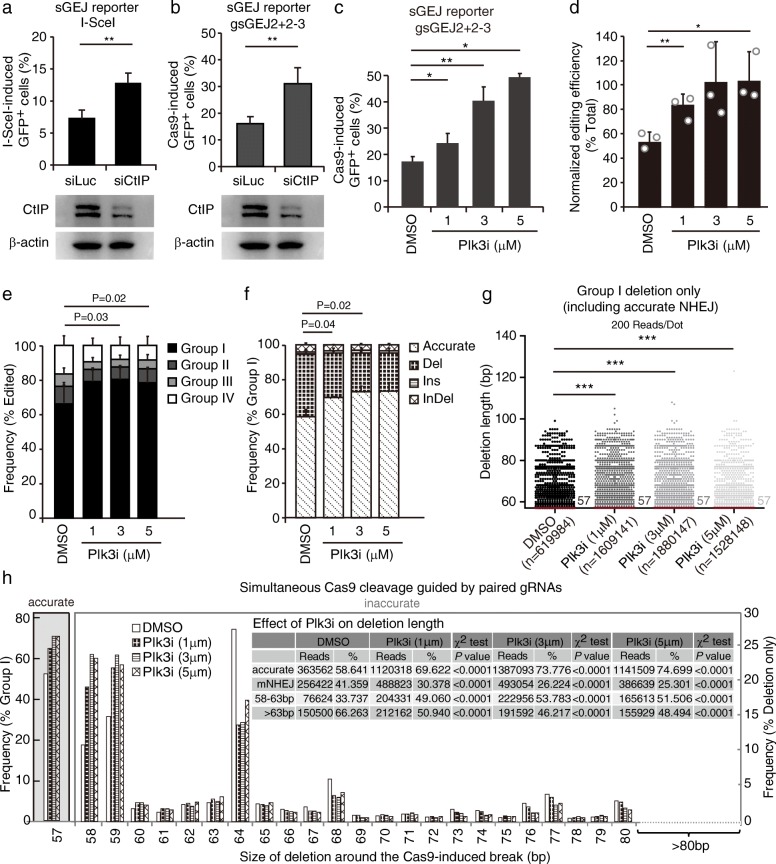
The Plk3 inhibitor GW843682X enhances accurate NHEJ in the repair of Cas9-induced DSBs. **a**, **b** Percentage of I-SceI- (**a**) or paired-Cas9- (**b**) induced GFP^+^ cells from sGEJ reporter cells transfected with siRNA against *CtIP* or Luciferase (*Luc*). Bars represent the mean ± standard deviation (SD) of three independent experiments, each performed in triplicate. In I-SceI-induced HR assays (**a**), Student’s paired *t*-test between siLuc and siCtIP **P = 0.0034; In Cas9-induced HR assays (**b**), Student’s paired *t*-test between siLuc and siCtIP **P = 0.0006. Depletion of *CtIP* was determined by western blotting using β-actin as a loading control. **c** Percentage of paired Cas9-induced GFP^+^ cells from sGEJ reporter cells treated with the Plk3 inhibitor GW843682X at concentrations of 1 μM, 3 μM, and 5 μM. Bars represent the mean ± SD of three independent experiments, each performed in triplicate. Student’s paired *t*-test between DMSO and 1 μM *P = 0.016; between DMSO and 3 μM **P = 0.003; between DMSO and 5 μM *P = 0.005. **d**–**f** Analysis of NHEJ induced by paired Cas9-gRNAs at the *LDHA* site in mouse ES cells treated with the Plk3 inhibitor at different concentrations. The normalized editing efficiency (**e**), the frequency of each group in edited events (**f**), and the frequency of each category in group I events (**g**) were calculated. Bars represent the mean ± SD of three independent experiments. For the normalized editing efficiency (**d**), Student’s paired *t*-test between DMSO and 1 μM **P = 0.006; between DMSO and 5 μM *P = 0.04. **g** Deletion length distributions of group I Del events at the *LDHA* site in mouse ES cells treated with the Plk3 inhibitor. The reads were combined from three independent experiments. Each *dot* represents 200 reads. The median deletion length is indicated, and deletion length distributions demonstrate a shift towards longer deletions in cells treated with the Plk3 inhibitor (Mann–Whitney test between DMSO and Plk3i ***P < 0.0001). **h** Frequency of accurate NHEJ among group I (*left*) and frequency of deletions with different deletion length in Del events of group I NHEJ (*right*) in mouse ES cells treated with Plk3i at different concentrations as indicated. Del NHEJ events were grouped into 58–63 bp and > 63 bp. The respective reads and frequencies are summarized in the inset and compared by a χ^2^ test with *P* values as indicated

As accurate NHEJ could improve the CRISPR/Cas9 genome editing that requires precise deletions of defined length, it is beneficial to find a method to enhance accurate NHEJ in this application. As CtIP-dependent end resection is a key step in NHEJ and tends to repress accurate end joining [39–41], we wondered whether inhibition of CtIP-dependent end resection could elevate accurate NHEJ. We first examined the effect of CtIP depletion on overall NHEJ using our mouse ES cells containing the sGEJ reporter [11]. Depletion of CtIP by siRNA increased both I-SceI- and Cas9-induced NHEJ (Fig. 7a, b). Previous studies have shown that CtIP-dependent end resection was activated via CtIP phosphorylation by polo-like kinase 3 (Plk3) and could be inhibited by the Plk3 inhibitor GW843682X [39, 42]. We thus treated the sGEJ reporter cells with GW843682X at concentrations of 1, 3, and 5 μM in our NHEJ assays and observed that Cas9-induced NHEJ was stimulated compared to the mock treatment with DMSO (Fig. 7c).

To investigate whether the editing efficiency and the frequency of accurate NHEJ were increased by GW843682X, we performed our reporterless NHEJ assay at the *LDHA* site with paired Cas9-gRNAs. The normalized editing efficiency was elevated by GW843682X (Fig. 7d). In the edited products, group I events were increased from 68.78% ± 12.72% with DMSO to 81.87% ± 8.48% with the inhibitor (Fig. 7e). Importantly, GW843682X promoted accurate NHEJ in group I (Fig. 7f). The enhancement in both overall editing and accurate NHEJ indicated that GW843682X could be used to improve the genome editing requiring precise deletions of defined length mediated by accurate NHEJ.

Detailed analysis of repair junctions further revealed that deletions were shifted towards shorter length in cells treated with GW843682X, although the median length remained at 57 bp in all treatments, including DMSO (Fig. 7g; Mann–Whitney test, *P* < 0.0001). Furthermore, GW843682X enhanced accurate NHEJ and reduced mutagenic NHEJ. The frequency of accurate NHEJ was elevated to 69.62%, 73.78%, and 74.70%, respectively, by 1, 3, and 5 μM GW843682X from 58.64% with DMSO (Fig. 7h and inset; χ^2^ test, *P* < 0.0001). However, treatment with GW843682X at 1, 3, and 5 μM promoted deletions of 58–63 bp (i.e., 1–6 bp + 57-bp pop-out) with the frequencies at 49.06%, 53.78%, and 51.51%, respectively, vs 30.74% with the DMSO treatment (Fig. 7h and inset; χ^2^ test, *P* < 0.0001). In contrast, the frequencies of mutagenic NHEJ over 63 bp (i.e., 6 bp + 57-bp pop-out) were reduced to 50.94%, 46.22%, and 48.49% by different concentrations of GW843682X from 66.26% with the DMSO control (Fig. 7h and inset; χ^2^ test, *P* < 0.0001). These results suggest that the Plk3 inhibitor GW843682X might inhibit CtIP-dependent end resection, promoting both overall NHEJ and accurate end joining in repair of Cas9-induced DSBs, and could be used to improve genome editing that requires precise deletions of defined length mediated by accurate NHEJ.

## Discussion

The NHEJ process was thought to be error prone partly due to end trimming by active DNA nucleases and polymerases prior to end ligation in mammalian cells [8–10]. Evolutionarily, end trimming is essential to deal with modified or incompatible ends of DSBs induced by ionized radiation and radiomimetic chemicals, ensuring ends are ligatable but inducing indels [8–10]. In CRISPR/Cas9-mediated genome editing, many applications are dependent upon repair of Cas9-induced DSBs by mutagenic NHEJ, which generates heterogeneous indels at repair junctions [7, 32]. This gave an impression that NHEJ is also error prone in repair of Cas9-induced DSBs. However, for directly ligatable ends, such as those generated by Cas9 as well as I-SceI, end trimming may be unnecessary and even intrinsically suppressed in mammalian cells [16]. As a result, accurate NHEJ may occur at high frequencies. Indeed, using the paired Cas9-gRNA approach that was able to distinguish accurate NHEJ from unedited targets, we detected a high frequency of accurate NHEJ in the repair of Cas9-induced DSBs, indicating that NHEJ is inherently accurate for repairing Cas9-induced DSBs.

In order to analyze NHEJ that includes accurate and mutagenic end joining, a common approach is to use artificial NHEJ reporter systems integrated in the genome [43, 44]. However, this approach requires reporters whose development and integration into the genome are laborious. Moreover, NHEJ analysis is restricted to the site on the reporter. As both accurate and mutagenic NHEJ can be analyzed and measured at intended sites in cells and in vivo by the paired Cas9-gRNA approach presented here, this provides an alternative but flexible and reporterless approach for NHEJ assays. This method was validated in *XRCC4*^*−/−*^ mouse ES cells and in mouse liver and could be further expanded to different genomic loci, organs, development stages, and genetic background for analysis of NHEJ, whether accurate or mutagenic.

In addition to frequent use of accurate NHEJ, joining distal ends of adjacently paired Cas9-induced DSBs has several other distinct features. First, + 1 and + 2 templated insertions occur frequently. Previous studies have shown that Cas9 generated mainly blunt ends but sometimes also ends with 5′-overhangs, especially 1- and 2-nt 5′-overhangs [3, 22, 31–33]. It was proposed that these 5′-overhanging ends could be filled in by DNA polymerases, resulting in templated insertions [31]. At a single Cas9-induced DSB, these 5′-overhanging ends are complementary and can be accurately rejoined without any fill-in by DNA polymerases. In contrast, distal ends with 5′-overhangs in paired DSBs may not be compatible for accurate NHEJ, leading to more frequent templated insertions and underestimation of accurate NHEJ in repair of single Cas9-induced DSB. However, by placing paired Cas9-gRNAs in the W/C orientation of the PAMs, not in W/W, C/W, and C/C, these frequent + 1 and + 2 templated insertions can be avoided. This provides a strategy to increase accurate NHEJ in genome editing.

Second, the distance between paired DSBs is flexible but has little effect on the occurrence of accurate NHEJ within the range of 23–148 bp. Prior work has indicated that the distance between paired DSBs influences the efficiency of accurate NHEJ when the distance is over 1 kb [22, 29, 30]. In this study, we focused on distances ranging from 23 to 148 bp with the exception of 1469 bp and detected little distance effect on accurate NHEJ. Because adjacently paired Cas9 on a target should not overlap with each other in order to prevent inhibition of simultaneous Cas9 cleavage, the minimum length of precise deletions by the paired Cas9-gRNA method could be 12 bp with the W/C orientation, 20 bp with C/C and W/W, and 34 bp with C/W.

Lastly, the frequencies of + 1 and + 2 templated insertions vary significantly at different genome sites. While this variation can be influenced by the orientations of the paired PAMs and possibly the distance between two joining ends, it can still dramatically differ even with the same PAM orientations and similar distance between paired DSBs. For example, at two sites of human *HPRT* locus (h*HPRT*#4 and #9; Additional file 1: Table S3), which carry the same W/W orientation and the same distance of 85 bp, one has a frequency of + 1 templated insertions without any additional mutations of 57.52% and the other 14.34%. This suggests that there exist additional but unknown determinants for highly frequent template insertions. Nevertheless, templated insertions may provide a target for improving the efficiency and the accuracy of CRISPR/Cas9-mediated genome editing.

As precise genomic deletions of defined length at a target are an important application in genome editing, a high level of accurate NHEJ would improve such an application. Previously, targeted genomic deletions via two concurrent DSBs have been used in generating disease models and developing gene therapies for human diseases as well as in studying coding and non-coding elements at individual genome loci or in a high-throughput fashion [4, 23, 45–57]. However, while these targeted genomic deletions were long and many were precise, accurate NHEJ was not systematically analyzed nor harnessed to improve these applications. The paired Cas9-gRNA method presented here can generate precise genomic deletions of defined length at high frequencies and has the capacity to increase the efficiency of gene knockouts and targeted in-frame deletions. The extent of the increase is usually higher in targeted in-frame deletions than in gene knockouts because the basal level is already high at about 66.7% in out-of-frame mutations even without engaging accurate NHEJ. Nevertheless, the heterogeneity of mutations is significantly reduced in both applications since the majority of deletions in these applications are precise deletions of defined “out-of-frame” or “in-frame” length.

This method can also be used for knock-in via precise blunt end ligation with exogenous DNA fragments in place of the excised sequence as done previously [17, 21, 58]. Similar to improvement of gene knockouts and targeted in-frame deletions, we could further improve the efficiency of precise knock-in by choosing the W/C orientation of paired PAMs to avoid frequent + 1 and + 2 templated insertions and by using the Plk3 inhibitor GW843682X to enhance accurate NHEJ. As the PAM compatibility is expanded, more sites at a target could be selected for the paired Cas9-gRNA approach, providing flexibility in choosing paired gRNAs according to the predefined deletion length, the PAM orientation, the efficiency of concurrent Cas9 cleavage, and even the efficiency of accurate NHEJ directly [24–26]. Expanded compatibility of PAM will further broaden applications of this method, which is a valuable addition to the CRISPR/Cas9 toolbox in genome editing.

## Conclusions

NHEJ is inherently accurate in the repair of Cas9-induced DSBs, leading to a high frequency of accurate NHEJ. This can be exploited by use of paired Cas9-gRNAs to improve genome editing that requires precise deletions of defined length, such as gene knockouts and targeted in-frame deletions. In this paired Cas9-gRNA method, the frequency of accurate NHEJ is hindered by frequent + 1 and + 2 templated insertions. Based on the findings, several rules have been devised to promote accurate NHEJ for genome editing (Additional file 2: Figure S11). These rules include preferentially selecting the W/C orientation of paired PAMs to prevent templated insertions, choosing the distance of predefined 3n + 2 bp between paired DSBs for efficient gene knockouts, and using the Plk3 inhibitor GW843682X to inhibit end resection by CtIP. With expanded compatibility of PAMs, applications of this strategy can be further broadened in genome editing and synthetic biology that require precise deletions of variable length. In addition, the paired Cas9-gRNA method provides a convenient, flexible, and reporterless approach to analyze both accurate and mutagenic NHEJ in cells and in vivo. By overcoming the limitations present in NHEJ reporters, this method can be used to analyze NHEJ at different loci and development stages and in different organs and genetic backgrounds.

## Methods

### Plasmids

Expression plasmids for gRNAs were constructed from the pU6-gRNA vector as described previously [28]. The Cas9 plasmid pX330 was originally obtained from Addgene (catalog number 42230). The pcDNA3β-based expression vectors for I-SceI and GFP were as described before [11]. The gRNA target sequences are listed in Additional file 1: Tables S5. Newly constructed plasmids were confirmed by Sanger sequencing.

### Cell lines

Mouse ES cells containing the sGEJ reporter, the BGN reporter, and the HR reporter were previously established and cultured as described before [11, 13, 28]. Isogenic *XRCC4*^*+/+*^ and *XRCC4*^*−/−*^ mouse ES cells containing the sGEJ reporter were also previously generated [11]. Human HEK293 and U2OS cells were cultured in DMEM containing 10% fetal bovine serum, 1% penicillin-streptomycin, and 2 mM L-glutamine. For Cas9-mediated *MDC1* knockout and *53BP1* targeted in-frame deletions, 2 × 10^5^ ES cells were transfected with the expression plasmids for paired gRNAs and Cas9 in a 24-well plate using Lipofectamine 2000 (Invitrogen) as previously described [28], and were seeded on mouse embryonic fibroblast (MEF) feeder cells for single clones without antibiotic selection in a 10-cm plate at 72 h post-transfection. In about 2 weeks, knockout clones and clones with targeted in-frame deletions were picked, grown, and verified by PCR along with Sanger sequencing and western blotting. Primers are listed in Additional file 1: Tables S5.

### Genome editing by paired I-SceI and paired Cas9-gRNAs

For genome editing by paired I-SceI, mouse ES cells harboring the sGEJ reporter or not were transfected with pcDNA3β-I-SceI. For genome editing by paired Cas9-gRNA, mouse ES cells and human HEK293 and U2OS cells were transfected with the expression plasmids for paired gRNAs and pX330 for Cas9. These cells were also transfected with pcDNA3β-GFP for transfection efficiencies. Transfection was done with Lipofectamine 2000 (Invitrogen) in 24-well plates as described previously [12]. At 72–96 h post-transfection, cells were harvested and genomic DNA was isolated for analysis of genome editing. For genome editing in vivo, wild-type male C57BL/6 mice, 6–8 weeks old, were purchased from Shanghai SLAC Laboratory Animal Co. Ltd. Hydrodynamic tail vein injection was performed as previously described with modifications [59]. Briefly, the expression plasmids for spCas9 and paired gRNAs (gRNA#1 and gRNA#2) targeting the *LDHA* sites were suspended in 1.8 mL saline and injected into mice in less than 10 s via the tail vein. The amount of injected DNA was 200 μg for Cas9, 150 μg for gLDHA#1, and 150 μg for gLDHA#2 for each mouse. At 30 days post-injection, liver tissues were harvested and genomic DNA was isolated for analysis of genome editing at the *LDHA* site.

### Genomic DNA extraction, PCR amplification, and Illumina deep sequencing

For analysis of targeted genome editing at endogenous genome loci, cells or liver tissues were collected after NHEJ of paired DSBs induced by paired I-SceI or paired Cas9-gRNAs. Genomic DNA was isolated from these cells or tissues using a genomic DNA purification kit (Axygen). The genomic regions targeted by I-SceI and CRISPR/Cas9 were PCR amplified with respective primers (Additional file 1: Table S5). PCR products were purified using a gel extraction kit (Axygen). PCR products of two to six different genomic target sites were then mixed, end-repaired, adenylated at 3′ ends, ligated with adapters, purified, and amplified by the second round of PCR to incorporate the P7 and P5 Illumina adapters and a unique 8-mer barcode sequence according to the manufacturer’s protocols. Next-generation sequencing was performed on the Illumina Hiseq at Veritas Genetics Asia Inc. (Hangzhou). Sequences were analyzed to identify edited events with different indels at repair junctions using DBS-Aligner as described previously [28].

### HR and NHEJ reporter assays

Mouse ES cells harboring the NHEJ or HR reporter were transfected with pcDNA3β-I-SceI, the pU6-gRNA plasmids and pX330, and/or siRNA as previously described [28]. siRNAs were purchased from RiboBio Co., and siRNA sequences were 5′-CGTACGCGGAATACTTCGA -3′ for the luciferase control and 5′-GGAACTCTGGACAAAACTA- 3′ for mouse CtIP. Both siRNAs were tested before in mouse ES cells [11]. For drug treatment, GW843682X (Sigma-Aldrich) was added at 6 h post-transfection and replaced fresh the next day for continued treatment for the rest of the experiment. Cells transfected and/or treated were analyzed for GFP^+^ frequencies using the Beckman Coulter CytoFLEX flow cytometer 3 days post-transfection. The NHEJ and HR frequencies were calculated after being corrected with background readings and normalized with transfection efficiencies as described before [11].

### Antibodies, western blotting, and immunofluorescence

Primary antibodies included anti-MDC1 (ab11169; 1:1000) and anti-53BP1 (ab21083; 1:1000) from Abcam, anti-CtIP (sc-271339; 1:1000) from Santa Cruz, anti-β-actin (A5441; 1:10000) from Sigma, and anit-γH2AX (2455636; 1:1000) from Millipore. For western blotting, cells were washed with cold PBS and lysed with RIPA buffer for 30 min. Cell extracts were separated by SDS-PAGE and analyzed by western blotting with corresponding antibodies. For immunofluorescence staining, cells were seeded on glass coverslips in six-well plates overnight and irradiated with 3 Gy X-rays. After 1-h recovery, cells were fixed with 4% paraformaldehyde, stained with primary antibodies and Alexa Fluor 488 or Alexa Fluor 546 secondary antibodies (Invitrogen), and imaged by the Zeiss AXIO Observer A1 microscope.

### Statistics

Data were analyzed by Student’s paired and unpaired *t*-test, one-way ANOVA with post-hoc least significant difference (LSD) pairwise comparisons, Kruskal–Wallis test, regression analysis, Mann–Whitney test, χ^2^ test for determination of *P* value and correlation coefficients as indicated in the figures.

## Additional files

Additional file 1: **Table S1.** The frequencies of accurate NHEJ in repair of Cas9-induced DSBs. **Table S2.** Top five group I NHEJ events in repair of adjacently paired DSBs induced by paired Cas9-gRNA. **Table S3.** Most of the + 1 insertions at repair junctions are templated insertions. **Table S4.** The effect of accurate NHEJ on the frequency of out-of-frame and in-fame editing. **Table S5.** Sequence information on gRNAs, primers, and genome editing target sites. (XLSX 216 kb)

Additional file 2: **Figure S1.** Comparison of accurate NHEJ in repair of Cas9-induced DSBs between human cells (HEK293 or U2OS) and mouse ES cells. **Figure S2.** The frequency of “Accurate”, “Deletion”, “Insertion” and “IndDel” in group I events from 71 endogenous gene loci. **Figure S3.** Nearly all of the + 1 insertions with no additional mutations at repair junctions were templated. **Figure S4.** Generation of + 1 templated insertions (TI) resulted from paired Cas9 cleavage with the *W*/W, W/C, C/W, or C/C orientation of paired PAMs. **Figure S5.** Nearly all of the + 1 templated insertions with no additional mutations could be predicted from paired Cas9 cleavage at the third and fourth base upstream of a PAM. **Figure S6.** Correlation between + 1 template insertions (*TI*) and out-of-frame mutations derived from either of the Common, Ideal, or Paired methods. **Figure S7.** Effect of accurate NHEJ with a frequency below or above 30% on the frequency of out-of-frame editing. **Figure S8.** Correlation between the out-of-frame editing frequency and the frequency of templated insertions (*TI*) at 50 genome sites. **Figure S9.** Comparison of the in-frame editing frequency induced by Common, Paired, and Ideal at 20 genome sites. **Figure S10.** Distributions of microhomology at repair junctions in *53BP1* wild-type, *53BP1*ΔTudor, and *53BP1*ΔOD mouse ES cells. **Figure S11.** Flowchart of the paired Cas9-gRNA protocol for genome editing that requires precise deletion of defined length. (PDF 4737 kb)
