# Supplementary material for: Harnessing accurate non-homologous end joining for efficient precise deletion in CRISPR/Cas9-mediated genome editing
Source: Genome Biol. 2018 Oct 19;19:170. doi: 10.1186/s13059-018-1518-x (PMC6195759; doi:10.1186/s13059-018-1518-x)
Supplement: Supplementary file 2 — Figure S1. Comparison of accurate NHEJ in repair of Cas9-induced DSBs between human cells (HEK293 or U2OS) and mouse ES cells. Figure S2. The frequency of “Accurate”, “Deletion”, “Insertion” and “IndDel” in group I events from 71 endogenous gene loci. Figure S3. Nearly all of the + 1 insertions with no additional mutations at repair junctions were templated. Figure S4. Generation of + 1 templated insertions (TI) resulted from paired Cas9 cleavage with the W/W, W/C, C/W, or C/C orientation of paired PAMs. Figure S5. Nearly all of the + 1 templated insertions with no additional mutations could be predicted from paired Cas9 cleavage at the third and fourth base upstream of a PAM. Figure S6. Correlation between + 1 template insertions (TI) and out-of-frame mutations derived from either of the Common, Ideal, or Paired methods. Figure S7. Effect of accurate NHEJ with a frequency below or above 30% on the frequency of out-of-frame editing. Figure S8. Correlation between the out-of-frame editing frequency and the frequency of templated insertions (TI) at 50 genome sites. Figure S9. Comparison of the in-frame editing frequency induced by Common, Paired, and Ideal at 20 genome sites. Figure S10. Distributions of microhomology at repair junctions in 53BP1 wild-type, 53BP1ΔTudor, and 53BP1ΔOD mouse ES cells. Figure S11. Flowchart of the paired Cas9-gRNA protocol for genome editing that requires precise deletion of defined length. (PDF 4737 kb) [file 13059_2018_1518_MOESM2_ESM.pdf]

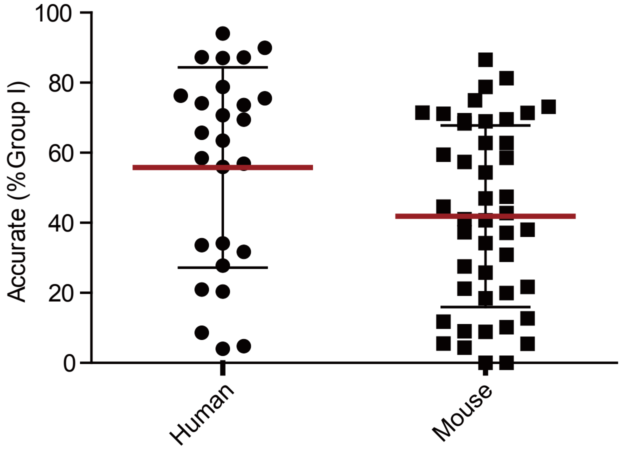

Figure S1. Comparison of accurate NHEJ in repair of Cas9-induced DSBs between human cells (HEK293 or U2OS) and mouse ES cells. 26 human endogenous genome sites and 44 mouse endogenous genome sites were edited by paired Cas9-gRNAs. The frequencies of accurate NHEJ in Group I events were analyzed.

Figure S2

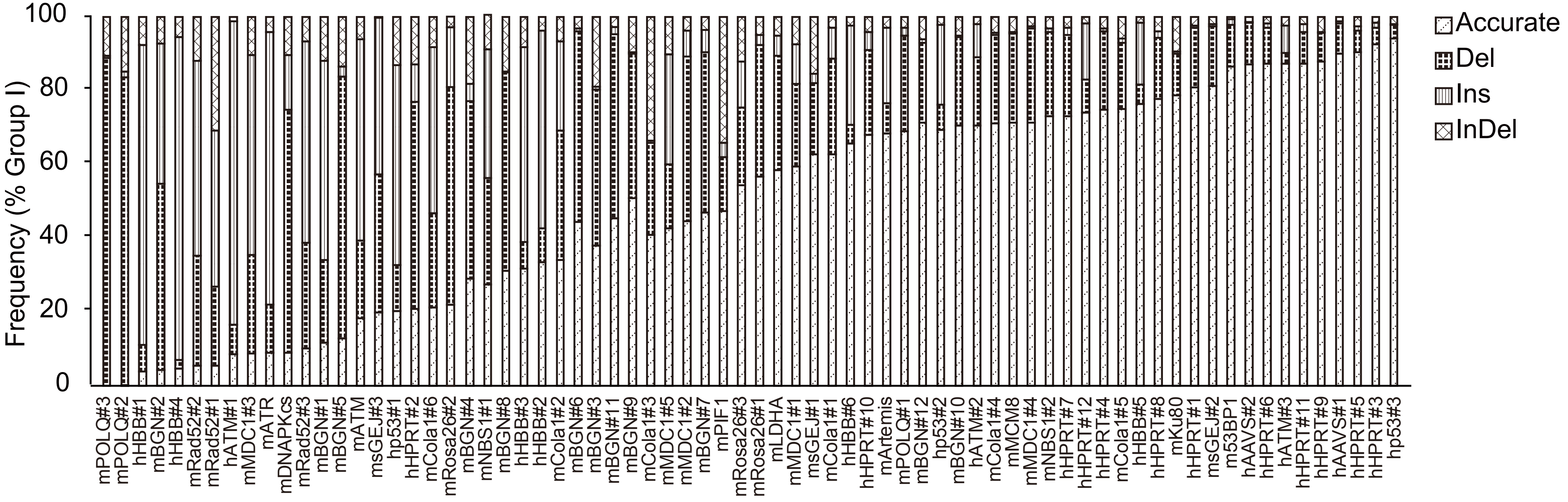

Supplementary Figure 2. The frequency of "Accurate", "Deletion", "Insertion" and "IndDel" in Group I events from 71 endogenous gene loci. The frequency of accurate NHEJ, deletion, insertion and InDel was calculated as ratios of reads from each category in Group I to total Group I reads.

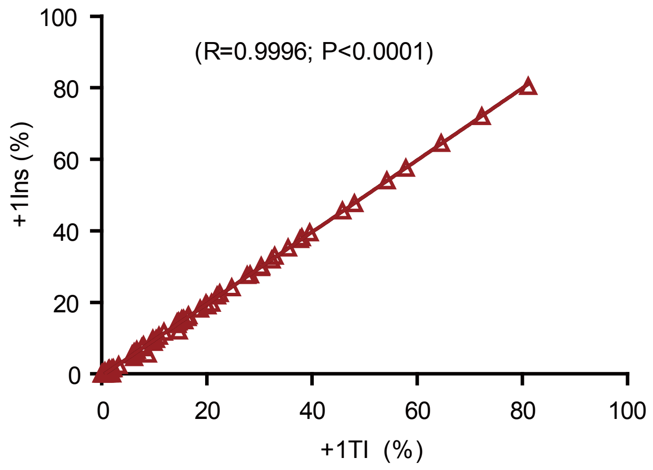

Figure S3. Nearly all of +1 insertions with no additional mutations at repair junctions were templated. +1 insertions and +1 templated insertions have nearly a perfect positive correlation as indicated. +1Ins: +1 insertions; TI: templated insertions.

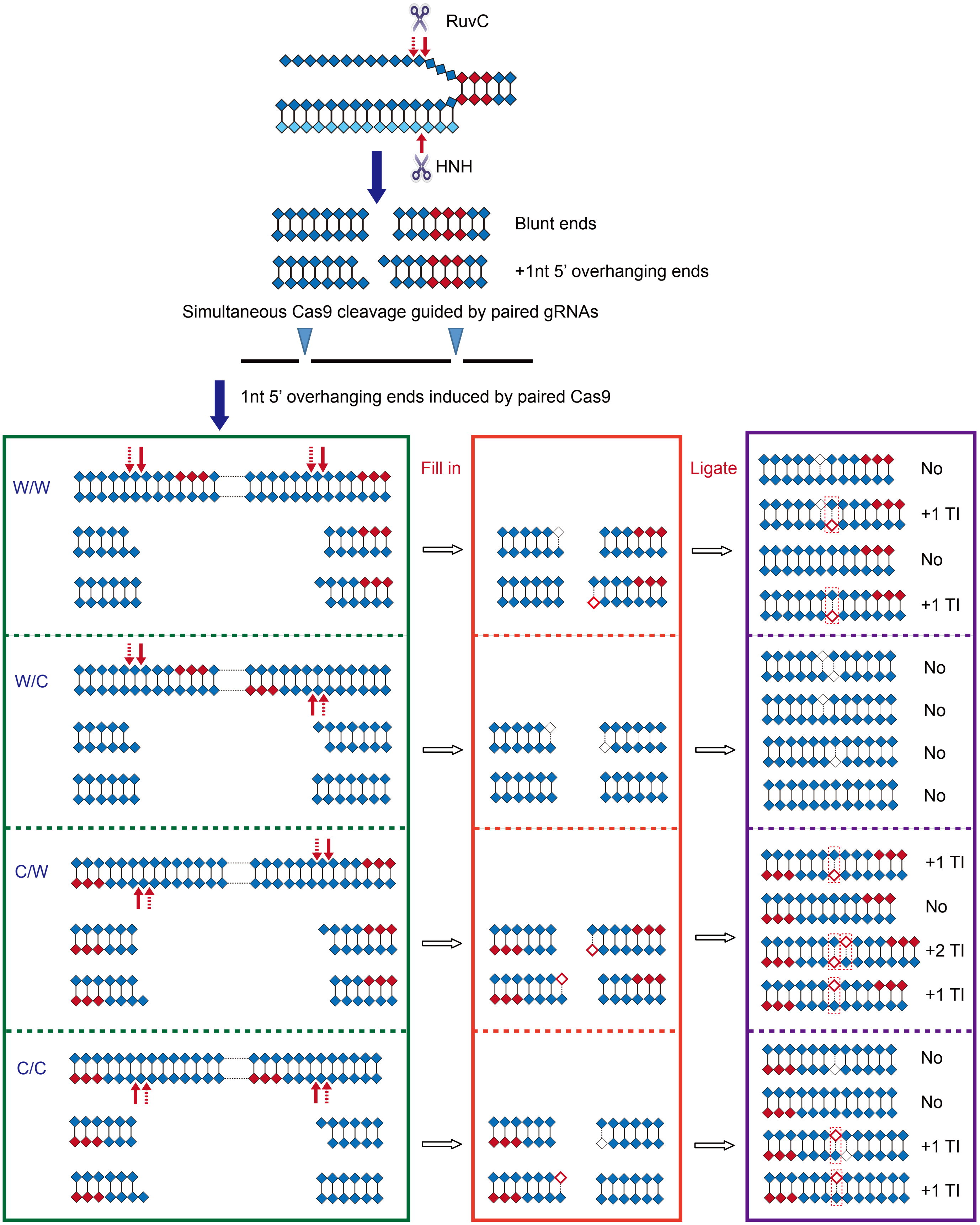

Figure S4. Generation of +1 templated insertions (TI) resulted from paired Cas9 cleavage with the W/W, W/C, C/W or C/C orientation of paired PAMs. The RuvC domain of Cas9 can cleave at the 3rd or the 4th base upstream of a PAM, generating not only blunt ends but also 1-nt 5'-overhanging ends. 1-nt 5'-overhanging ends are filled in with 1-nt by DNA polymerases. The 1-nt fill-in resulting in +1 templated insertions is indicated in red empty diamond shape and the other 1-nt fill-in resulting in no templated insertions in empty diamond shape. We predict that W/W and C/C give rise to frequent +1 templated insertions with no additional mutations, while C/W generate +1 and +2 templated insertions with no additional mutations and W/C no such templated insertions as indicated.

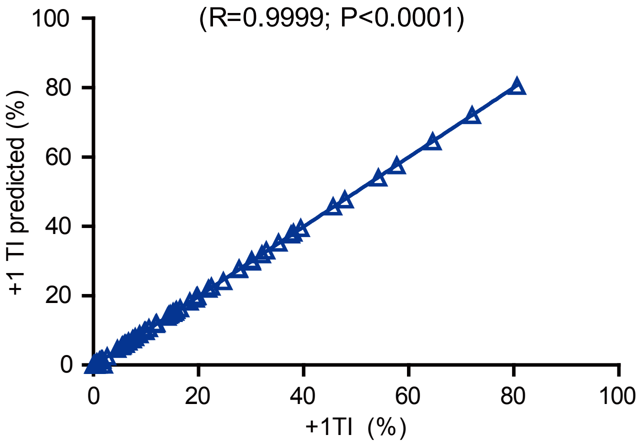

Figure S5. Nearly all of +1 templated insertions with no additional mutations could be predicted from paired Cas9 cleavage at the third and fourth base upstream of a PAM. +1 templated insertions predicted have nearly a perfect positive correlation with actual +1 templated insertions as indicated. TI: templated insertions.

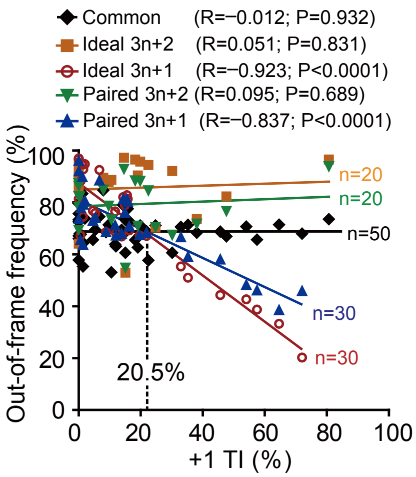

Figure S6. Correlation between +1 template insertions (TI) and out-of-frame mutations derived from either of the "Common", "Ideal" and "Paired" methods. Common: black rhombus; Ideal 3n+2: orange square; Ideal 3n+1: red circle; Paired 3n+2: green triangle; Paired 3n+1: blue triangle. "3n+1" and "3n+2" indicate predefined length in base pairs for precise deletions mediated by accurate NHEJ in each approach.

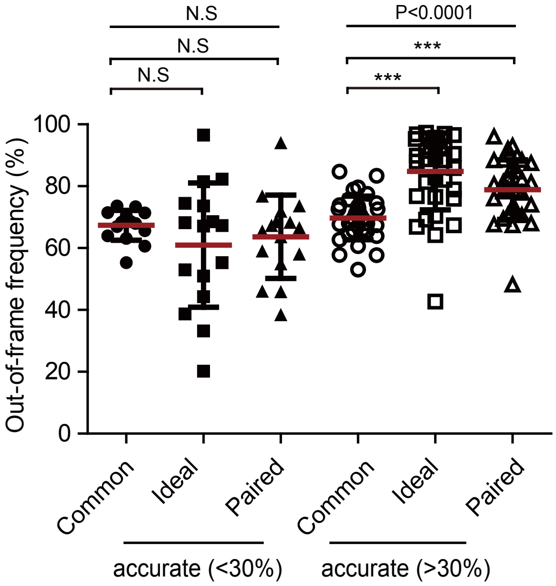

Figure S7. Effect of accurate NHEJ with a frequency below or above 30% on the frequency of out-of-frame editing. Accurate NHEJ with a frequency over 30% in the “Ideal” and “Paired” method increases the frequency of out-of-frame editing as compared to the “Common” approach. P values were determined by One-Way ANOVA with post hoc Least Significant Difference (LSD) test. ***: P≤0.0005; NS: not significant (i.e. P>0.05).

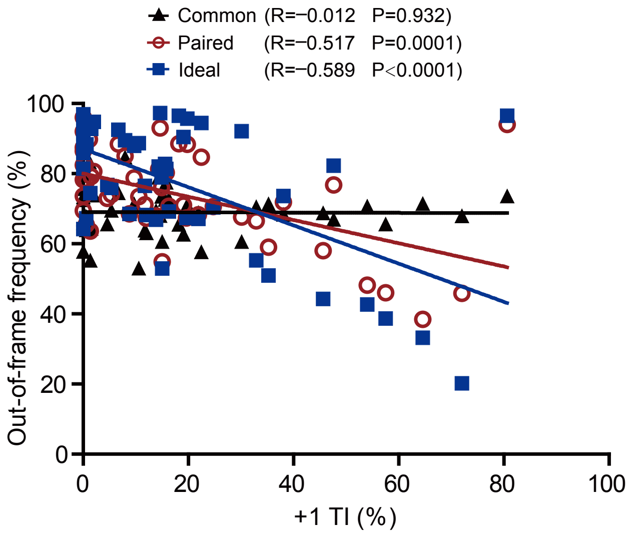

Figure S8. Correlation between the out-of-frame editing frequency and the frequency of templated insertions (TI) at 50 genome sites. The frequency of +1 templated insertions is negatively correlated with the out-of-frame editing frequency in the "Paired" and "Ideal" method, not in the "Common" method. Statistics were performed using regression analysis.

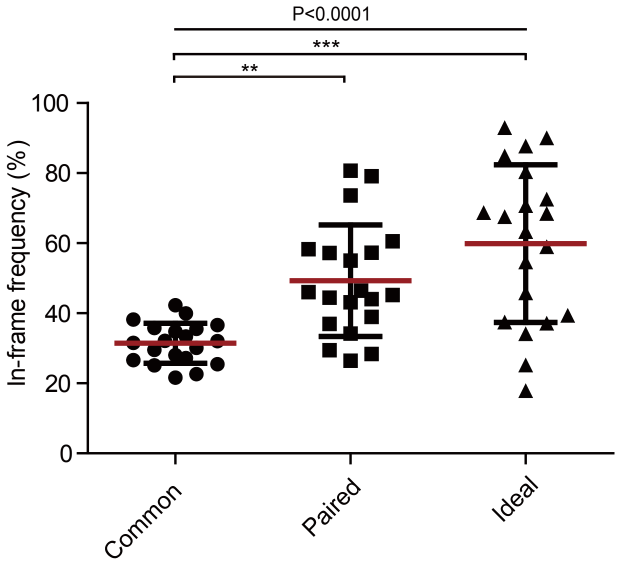

Figure S9. Comparison of the in-frame editing frequency induced by "Common", "Paired" and "Ideal" at 20 genome sites. The in-frame editing frequency is higher in the "Paired" or "Ideal" method than in the "Common" method. P values were determined by One-Way ANOVA with post hoc LSD test. ****: P≤0.0001; ***: P≤0.0005.

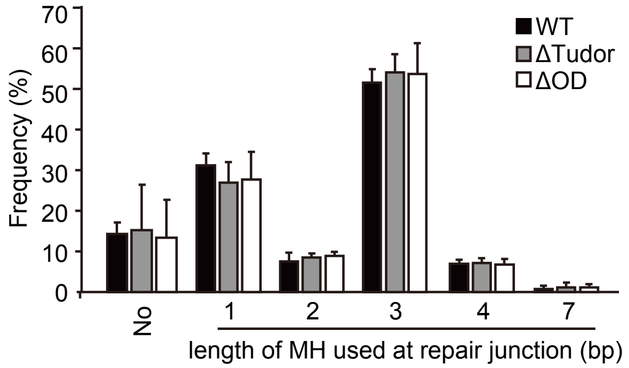

Figure S10. Distributions of microhomology at repair junctions in *53BP1* wild-type, *53BP1*ΔTudor and *53BP1*ΔOD mouse ES cells. WT: wild-type; MH: microhomology.

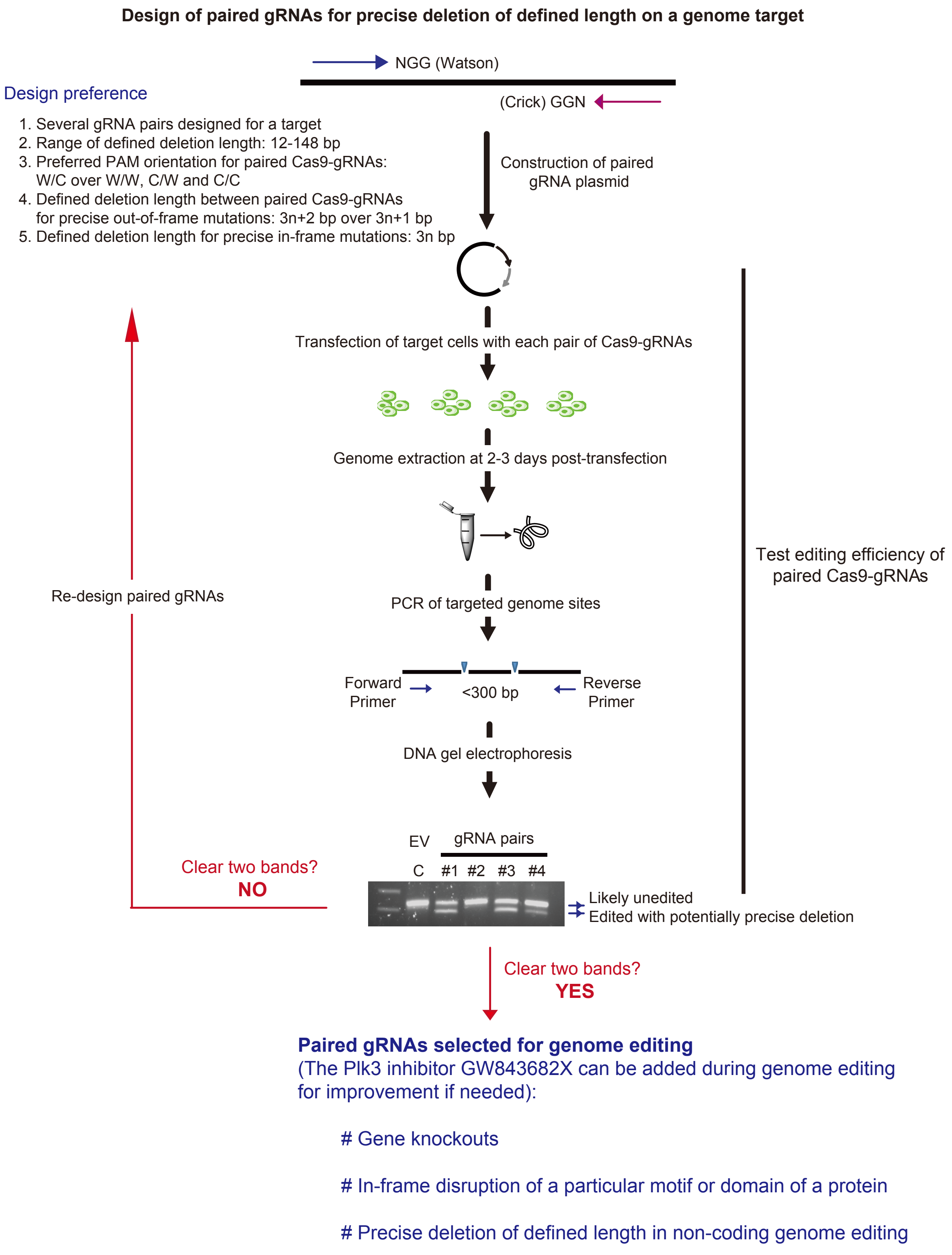

Figure S11. Flowchart of the paired Cas9-gRNA protocol for genome editing that requires precise deletion of defined length. C: gRNA empty vector (Control).
